# A biophysical model for plant cell plate maturation based on the contribution of a spreading force

**DOI:** 10.1093/plphys/kiab552

**Published:** 2021-11-27

**Authors:** Muhammad Zaki Jawaid, Rosalie Sinclair, Vincent Bulone, Daniel L Cox, Georgia Drakakaki

**Affiliations:** 1 Department of Physics and Astronomy, University of California, Davis, California, USA; 2 Department of Plant Sciences, University of California, Davis, California, USA; 3 School of Food, Agriculture and Wine, The University of Adelaide, Waite Campus, Adelaide SA 5064, Australia; 4 Department of Chemistry, Division of Glycoscience, KTH Royal Institute of Technology, Stockholm, Sweden

## Abstract

Plant cytokinesis, a fundamental process of plant life, involves de novo formation of a “cell plate” partitioning the cytoplasm of dividing cells. Cell plate formation is directed by orchestrated delivery, fusion of cytokinetic vesicles, and membrane maturation to form a nascent cell wall by timely deposition of polysaccharides. During cell plate maturation, the fragile membrane network transitions to a fenestrated sheet and finally a young cell wall. Here, we approximated cell plate sub-structures with testable shapes and adopted the Helfrich-free energy model for membranes, including a stabilizing and spreading force, to understand the transition from a vesicular network to a fenestrated sheet and mature cell plate. Regular cell plate development in the model was possible, with suitable bending modulus, for a two-dimensional late stage spreading force of 2–6 pN/nm, an osmotic pressure difference of 2–10 kPa, and spontaneous curvature between 0 and 0.04 nm^−1^. With these conditions, stable membrane conformation sizes and morphologies emerged in concordance with stages of cell plate development. To reach a mature cell plate, our model required the late-stage onset of a spreading/stabilizing force coupled with a concurrent loss of spontaneous curvature. Absence of a spreading/stabilizing force predicts failure of maturation. The proposed model provides a framework to interrogate different players in late cytokinesis and potentially other membrane networks that undergo such transitions. Callose, is a polysaccharide that accumulates transiently during cell plate maturation. Callose-related observations were consistent with the proposed model’s concept, suggesting that it is one of the factors involved in establishing the spreading force.

## Teaser

The late-stage onset of an “areal” spreading and stabilizing force is essential for regular plant cell plate development and maturation.

## Introduction

**Figure 1:**
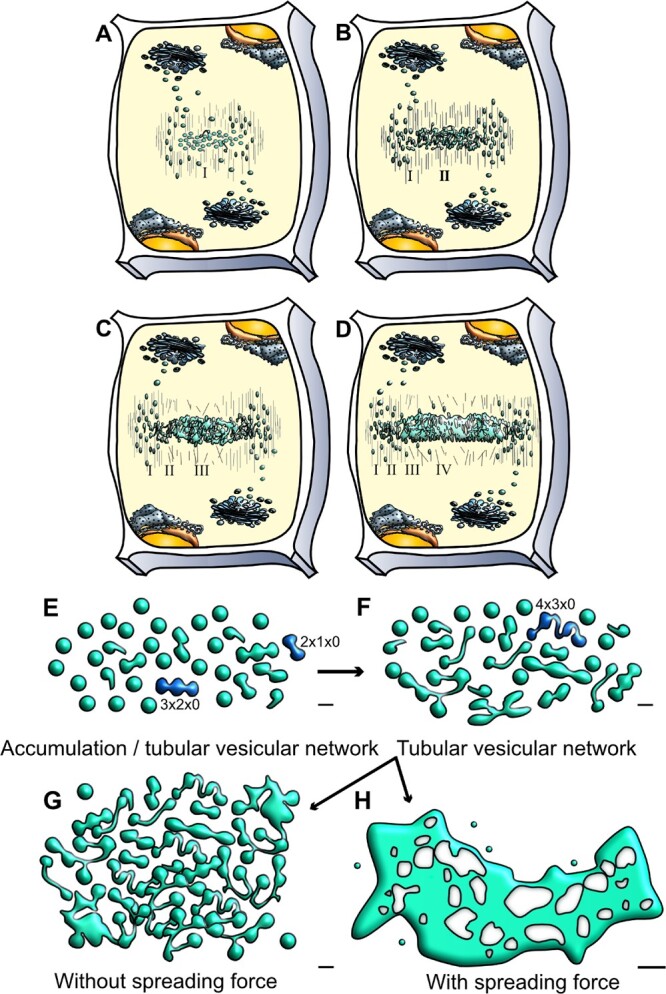
Schematic representation of cell plate development stages and the potential role of a spreading force in cell plate maturation. A–D, Cell plate development occurs centrifugally in multiple stages. A, During the first stage (I), cytokinetic vesicles guided by the phragmoplast accumulate at the center of the dividing cells, at the cell plate assembly matrix. B, Vesicles undergo fusion and fission and conformational changes resulting in TVN (Stage II). C, Interconnected membrane structures transition to a TN. At this stage high callose deposition occurs (Stage III). D, The membrane network further expands to an almost continuous fenestrated membrane sheet (PFS) (Stage IV). Deposition of additional polysaccharides helps transition to a new cell wall, separating the two daughter cells. Note that different stages can occur simultaneously, images are not to scale. This simplified representation emphasizes on cell plate membranes (Samuels et al., 1995; Segui-Simarro et al., 2004). E–H, Schematic representation of cell plate development describing the role of a spreading force. E, Early stages of vesicle accumulation and fusion and F, TVN and TN structures are shown. Two different possibilities are projected for stage transition (1) Incomplete/arrested cell plate G. In the absence of a spreading force G, tubular and fenestrated structures accumulate, and there is a lack of maturation towards a single, complete cell plate structure. (2) Normal cell plate transition H. In our calculations, we discover that for expansion/maturation to occur as in D, the presence of a spreading force is required, along with the decrease of spontaneous curvature to a threshold value. This allows for a sheet-like cell plate (SCP) structure to form. The structures in this schematic description are adapted from data collected from EM tomography (Segui-Simarro et al., 2004) with bars in E–G = 50 nm, H = 0.25 µm. Dark blue vesicles denote those labeled by the mathematical naming schema as described in Figure  2. Whereas in E, 2 × 1 × 0 denotes two oblate spheroids, one tubular connection, and zero holes.

Cytokinesis is a fundamental process of plant life that is different from animal cell cytokinesis. In plants, formation of a cell plate develops into the new cell wall, partitioning the cytoplasm of the dividing cell. Cell plate formation involves highly orchestrated vesicle accumulation, fusion, and membrane transformation concurrent with the time-specific deposition of polysaccharides such as callose, cellulose, and cross-linking glycans along with glycoproteins (Figure  1). This development requires choreographed accumulation of post-Golgi vesicles via the phragmoplast, an assembly of microtubules and microﬁlaments that help organize vesicle delivery to the cell plate assembly matrix, at the division plane (Lee and Liu, 2013).

Cell plate expansion is centrifugal, led by the accumulation and fusion of cytokinetic vesicles to the leading edge and maturation of the membrane network from the center (Samuels et al., 1995; Segui-Simarro et al., 2004; Lee and Liu, 2013; Smertenko et al., 2017). Cell plate development takes place in morphologically deﬁned stages (Figure  1). It starts with initial fusion of Golgi vesicles stage, in which cytokinetic vesicles are guided by the phragmoplast to the cell plate assembly matrix (Figure  1A). Fused vesicles are transformed into dumbbells that undergo conformational changes to form a tubulo-vesicular network (TVN) (Figure  1B), which transitions to a tubular network (TN) (Figure  1C). The TN expands into a planar fenestrated sheet (PFS). As the gaps in the fenestrated sheet are gradually closing, this leads to the formation of the young cell wall sandwiched between two parallel plasma membranes that fuses with the parental cell wall (Figure  1D) (Samuels et al., 1995; Segui-Simarro et al., 2004). Excess membrane material is recycled concurrently, along with the accumulation of different polysaccharide materials. Based on elegant electron tomography studies, it is estimated that 70% of membrane material is removed during the transition of the cell plate from TVN to TN and PFS (Segui-Simarro et al., 2004). Analysis of vesicle dynamics support electron microscopy studies showing an initial vesicle delivery with fast expansion, followed by slower expansion phase (van Oostende-Triplet et al., 2017). It is notable that the multiple stages exist simultaneously (Figure  1), adding complexity in dissecting them (Drakakaki, 2015).

During cell plate expansion and maturation, membrane remodeling and network expansion are highly coordinated with the deposition of polysaccharides, providing an opportunity to study membrane morphology changes. The molecular basis of vesicle delivery at the cell plate has been extensively studied (McMichael and Bednarek, 2013; Boruc and Van Damme, 2015; Jurgens et al., 2015) with key components such as RAB GTPases, soluble *N*-ethylmaleimide-sensitive factor attachment protein receptor, tethering complexes, dynamin rings, and accessory proteins receiving attention (McMichael and Bednarek, 2013; Jurgens et al., 2015; Smertenko et al., 2017). However, the factors contributing to stage transition from a vesicular network to a fenestrated sheet, leading to cell plate maturation, are largely unknown. Dynamin rings and clathrin coated vesicles contribute to recycling of excess material (McMichael and Bednarek, 2013), while the deposition of polysaccharides likely contributes to transition into a mature cell plate. Hemicelluloses and pectins are deposited via Golgi derived vesicles. Callose and cellulose are directly synthesized at the cell plate (Miart et al., 2014; Chen et al., 2018). Callose, a β-1-3 glucan is a dominant polysaccharide transiently synthesized at the cell plate. Structural glycoproteins such as extensins are part of the newly formed cell plate (Cannon et al., 2008) and can contribute to cell plate maturation. Given the complexity of cell plate development and the concurrent presence of different stages, a biophysical model can be used as a framework for interrogation of individual components that can provide insights and guide future research.

In this study, we used biophysical modeling to dissect the transition between the vesicular network stage to a fenestrated sheet and a mature cell plate. We tested the hypothesis that a time-dependent spreading and stabilizing force is necessary for cell plate maturation. We could model this force by adding a phenomenological “areal pressure” term to the Helfrich model free energy for the cell plate surface. Furthermore, we monitored its influence by adopting a variational approach to locally minimize the model free energy in time, assuming the process is sufficiently slow to consider the system close to thermodynamic equilibrium. The quasi-equilibrium is constantly redeﬁned as vesicles are added at the cell plate boundary. This enables us to use the total cell plate surface area as a proxy for time. We demonstrate semi-quantitatively that by assuming a late time onset of this spreading and stabilizing force, followed by the reduction of membrane spontaneous curvature, we can reproduce the observed morphological time dependent transition of the cell plate morphology.

## Results

We took a modeling approach to generate tools to dissect better membrane network transition during cell plate maturation. Due to the complexity of cell plate development, we decided to look for energy minima within a parameterized restrictive geometry basis set, thereby adopting a restricted variational approach within testable approximated structures. We found that existing general adaptive mesh approaches, such as Surface Evolver, while in principle more accurate, were not amenable for application in our study, due to their inability to incorporate the spreading/stabilizing force into such a large-scale system (Brakke, 1996).

### Shape approximation

**Figure 2:**
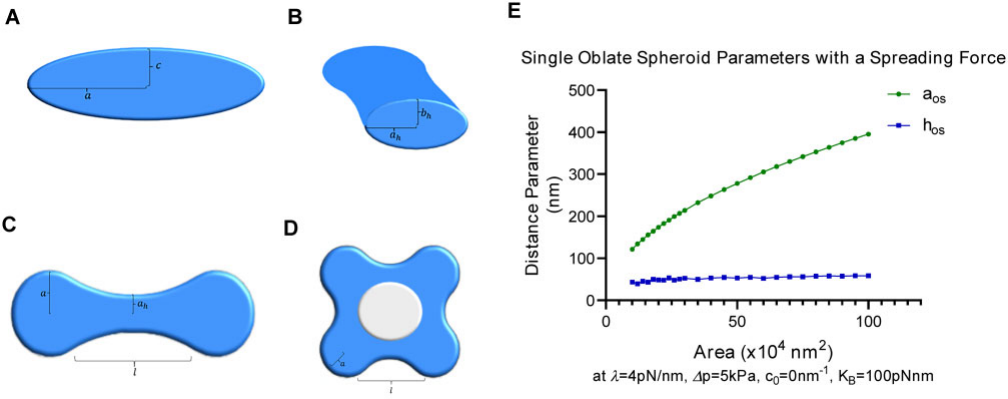
Approximating cell plate structures using a variational approach. A–D, Examples of membrane structure parameterizations used for modeling. A, Cross-section of an oblate spheroid through the polar axis. The major axis radius is labeled a and the minor axis radius is labeled c. This structure is used to model vesicles, or mature cell plate structures in the case where a≫c. B, Cross-section of an elliptic hyperboloid at its center, showing the skirt radii. The hyperboloid can be parameterized by its length l and its skirt radius in the equatorial plane $a_{h}$, the skirt radius in the axial plane is given by $b_{h}$, which can be written as a function of the other parameters listed as shown in Supplemental Equation S3. C, An example of a tubulo-vesicular structure parameterized by two oblate spheroids connected by a single elliptic hyperboloid (referred to as a 2 × 1 × 0 structure). Only the top view is shown. D, An example of a 4 × 4 × 1 conformation that models a transition to a fenestrated network with genus g = 1 (one gap). E, Evolution of single oblate spheroid parameters in the presence of a spreading force. In the presence of a spreading force, the thickness of the oblate spheroid remains in the 40–80 nm range despite the increase in area. This reflects the thicknesses and growth patterns found in intermediate cell plate stages (Samuels et al., 1995). Here, $h_{\mathrm{os}}\;$ =  2c ($a_{\mathrm{os}},\;c$ shown in A), represents the overall height, or thickness, of the oblate spheroid. In the absence of a spreading force, $h_{\mathrm{os}}$, or the thickness, is estimated to grow in values that are not observed experimentally. For reference, an area of $10^{4}nm^{2}$ is roughly equal to that of a single vesicle.

First, we approximated subcellular structures with testable shapes that could be used in a model. As shown in Figure  1, the cell plate, during its different transitional stages, contains cytokinetic vesicles, fused vesicles stretched to dumbbells, TVNs and fenestrated structures that finally mature to a complete cell plate and a new cell wall. These structures can be modeled using a combination of oblate spheroids and elliptical hyperboloids (Figures  1 and 2). Namely, cytokinetic vesicles can be approximated using oblate spheroids, where the two deﬁning radii can be used as variational parameters as shown in Figure  2A. The oblate spheroid can also be used to model the expanded/late-stage cell plate close to completion, as a very large oblate spheroid with a≫c.

Similarly, structures found within the fenestrated sheet and the TVN stages can be approximated using a combination of elliptic hyperboloids and oblate spheroids, such that the hyperboloids are continuous at the oblate spheroid boundaries. These elliptic hyperboloids can be parameterized by their length, hereafter referred to as l, and their skirt radius in the equatorial plane, hereafter referred to as $a_{h}$. The other parameters needed to deﬁne an elliptic hyperboloid can be written as a function of these two parameters due to boundary conditions that arise from mandating continuity. Figure  2B shows a cross-section of an elliptic hyperboloid, while Figure  2C shows an example of two vesicles joined by a single tube.

### Naming convention of different approximated conformations

The naming convention for different conformations follows the example: A shape that is labeled as 6  × 7 × 2, represents a conformation that has six oblate spheroids (or vesicles), seven hyperboloids (or tubes), and two gaps (or fenestrations, with g = 2). A 2 × 1 × 0 conformation, can approximate dumbbell structures found in early TNs, where two vesicles join via a single tube (Figure  2C). An example of a 4 × 4 × 1 conformation with a single fenestration, which can be used to approximate early fenestrated structures, is shown in Figure  2D.

### Energy minimization

The area of any given conformation was calculated by numerical integration methods, and a corresponding parameter space of a given area was found. For simplicity, we analyzed conformations that shared the same parameters for each of the oblate spheroids, and each of the hyperboloids with examples shown in Figure  2. We found a four-dimensional parameter space ($a,c,a_{h},l$) that corresponded to a given area up to an error tolerance (<0.01%) for each conformation of interest, and then calculated the energies of Equation 1 within that parameter space. The energy minimum was then extrapolated from that parameter space. Additional information about the four-dimensional parameter space as well as the calculation of the area elements used in the numerical integration methods is given in the supplemental information (Supplemental Eq. S1–S4, S10 and S11), and the full list of the parameters involved are shown in Supplemental Figure S1.

### Modified Helfrich energy

In order to identify contributing factors for cell plate maturation beyond the vesicle network stage, we modeled the free energy for the cell plate surface by adopting the Helfrich energy (Helfrich, 1973) with the addition of a novel term to model the presence of a spreading/stabilizing force. The free energy is deﬁned as follows:

(1) $$E\;=\;E_{\mathrm{bending}\;}+\;E_{\mathrm{pressure}}\;+\;E_{\mathrm{tension}\;}+\;E_{\mathrm{gaussian}\;}+\;E_{\mathrm{spreading}}\;$$

Each of the components is described in detail in the supplemental information corresponding to Supplemental Equations S5–S9.

The terms describe (1) the bending energy over the closed membrane surface(s) of the cell plate, which depends on the local curvature or the spontaneous curvature of the membrane, given by $c_{o}$, and the bending modulus, given by${\;K}_{B}$, (2) the pressure energy which results from the difference in osmotic pressure between the inside and the outside of the cell plate, represented by Δp, (3) the energy associated with the surface tension of the membrane, which depends on the local surface tension, given by γ, (4) the Gaussian bending energy, and (5) the novel term of spreading/stabilizing force.

The novel spreading force term is analogous to a two-dimensional pressure acting against the periphery of the cell plate structure along the equatorial plane. It is dependent on λ, which parameterizes the spreading/stabilizing force, having units of force/length. We allow for λ to be time-dependent, which would represent the “turning on” of polymer production in an expanding plate. We also allow for $c_{o}$ to be time-dependent, accounting for differences in spontaneous curvature that may arise from changes in membrane composition during cell plate evolution.

This methodology can also be used to provide a basis for the quantitative assessment of membrane structures found in the endoplasmic reticulum and the Golgi apparatus which are so far limited in a large-scale view using Helfrich theory. Earlier work (Shemesh et al., 2014) examined possible morphologies as a function of curvature modifying proteins using full minimization of the free energy via the Surface Evolver finite element approach (Brakke, 1996). However, these finite element methods were unable to consider the spreading/stabilizing force required in Supplemental Equations S6–S9 in any such available code. To establish a testable and functional model, we adopted the variational approach including multiple connected surfaces with negative curvature tubulations as a reasonable compromise approach to explore the quasi-equilibrium stabilities of different morphologies that are fully representative of the observed structures.

### Model parameter ranges and the need of a spreading/stabilizing force

We ﬁrst minimized the modified Helfrich energy (Eq. 1) for multiple conformations (vesicular, tubular, and fenestrated) to determine a range of parameters that would match the experimentally observed cell plate sizes/thicknesses. From electron tomography cryo-EM images of developing cell plates, we determined that the thickness of a cell plate in various stages of development was ∼40–120 nm (Segui-Simarro et al., 2004). Therefore, we tuned the free parameters in our energy model such that conformations’ thickness across the equatorial plane was in the range of 40–120 nm. We determined that, depending on the choice of the bending modulus, the allowed values of the spreading/stabilizing force parameter λ should be between 0.0 and 6.0 pN/nm, the spontaneous curvature $c_{o}$ between 0 and 0.04 $nm^{-1}$, and a ﬁnite pressure difference Δp around 2–10 kPa. A deviation from these ranges results in structures that are either too thick or too thin to exist in intermediate stages of cell plate development based on literature. An example of how we tuned the parameters to fit the experimental sizes and shapes is given in Figure  2E, in which a single oblate spheroid evolves with the area increase with given parameter values while maintaining the experimentally observed thickness. It is notable that a spreading force is necessary to achieve the desired values. A summary of the full range of parameter values are given in Table  1 and a full description of these parameters is provided in the supplemental information.

**Table 1:** Model parameter ranges

Parameter	Value
Bending modulus $K_{B}$	62.5–200 pNnm
Spontaneous curvature $c_{o}$	0–0.04 $nm^{-1}$
Pressure difference Δp	2–10 kPa
Spreading force parameter λ	2–6 pN/nm
Surface tension parameter γ	1.6 pN/nm
Gaussian bending modulus $K_{G}$	−0.8 $K_{B}$

The terms describe (1) the bending energy over the closed membrane surface(s) of the cell plate, which depends on the local curvature or the spontaneous curvature of the membrane, given by $c_{o}$, and the bending modulus, given by${\;K}_{B}$, (2) the pressure energy which results from the difference in osmotic pressure between the inside and the outside of the cell plate, represented by Δp, (3) the energy associated with the surface tension of the membrane, which depends on the local surface tension, given by γ, (4) the Gaussian bending energy, and (5) the term of spreading/stabilizing force.

Although the range of values for the pressure difference and the planar spreading force parameter were phenomenologically determined, they are within reasonable bounds. For instance, the solute concentration difference between the interior and the exterior of the cell plate by employing the van’t Hoff equation, which yields a solute concentration difference between 8 × 10–4 mol/l and 4 × 10–3 mol/l, comparable to protein solute concentration differences in higher plant cells. The spreading force required for cell plate maturation over a length of a nanometer is around 2–6 pN, which is comparable to the polymerization ratchet forces of a microtubule (Kent and Lele, 2017).

### A spreading force is required for cell plate maturation while its absence energetically favors the accumulation of tubular and vesicular networks

**Figure 3:**
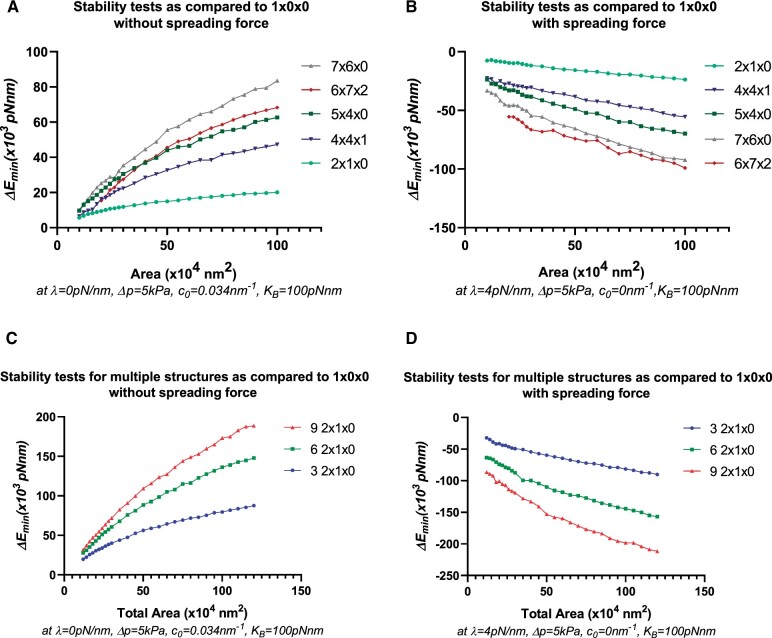
Stability tests to determine the role of a spreading force in different shape conformations. A and B, Stability tests determined by $\Delta E_{\min}$ versus Area for different conformations compared to a single oblate spheroid at the labelled area. A positive value of $\Delta E_{\min}$ indicates relative stability of the labelled conformation as compared to a single oblate spheroid (1 × 0 × 0). A, Relative stability of tubular (2 × 1 × 0, 5 ×4 × 0, 7 × 6× 0) and fenestrated (4 × 4 × 1, 6 × 7 × 2) structures in the absence of a spreading force with a finite spontaneous curvature. B, Stability of a single oblate spheroid over tubular and fenestrated structures in the presence of a spreading force and with zero spontaneous curvature. Note that in (B) a decrease of spontaneous curvature to a threshold value close to 0.015 $nm^{-1}\;$yields similar results. C and D, Stability test for multiple 2 × 1 × 0 structures compared to a single oblate spheroid at the labeled area. C, Relative stability of multiple 2 × 1 × 0 structures compared to a single oblate spheroid in the absence of a spreading force. At a labeled area, a larger number of structures have collectively a higher, more positive value of $\Delta E_{\min}$, thereby indicating that in the absence of a spreading force, tubular, as well as emerging fenestrated/network structures (as inferred by the results of A and B) are energetically favorable and tend to accumulate as shown in Figure  1G. D, Stability of a single oblate spheroid compared to multiple 2 × 1 × 0 structures in the presence of a spreading force and with zero spontaneous curvature. In the presence of a spreading force, at a labeled area, a larger number of structures have a lower, more negative value of $\Delta E_{\min}$ collectively, thereby indicating the energetic favorability of structures fusing to form larger, more mature structure(s).

Our goal was to assess within the modeled free energy of Eq. (1), whether a spreading force is essential for the necessary transitions from a combination of TVN to a fenestrated sheet and finally to a single mature cell plate structure. Applying Equation 1, we compared the energy minima compared to that of a mature cell plate (Figure  3A). From an energy perspective, we identified that in the absence of a spreading force, tubulo-vesicular and fenestrated structures have a lower value of energy at minima and are more stable than a single late-stage cell plate structure (resembled by a single oblate spheroid) of the same area. Figure  3A shows the energy minima of tubular and fenestrated structures (7 × 6 × 0,…) compared to those of a single oblate spheroid (1 × 0 × 0). $\Delta E_{\min}$ represents the difference of the energy minima value of the labelled structure with that of a single oblate spheroid, so that $\Delta E_{\min}$(2 × 1 × 0)  = $\;E_{\min}$(1 × 0 × 0)$-E_{\min}$(2 × 1 × 0). Thus, positive $\Delta E_{\min}$ indicate the relative stability of the labelled conformation. Our simulations indicate that in the absence of a spreading force, increased tubularity is preferred with the increase in area (Figuer 1G and 3A). Furthermore, in the absence of a spreading force, some fenestrated structures (4 × 4 × 1, 6 × 7 × 2) are also energetically stable and are therefore likely to accumulate.

We then examined the possibility of a transition from TVNs to a single oblate spheroid or a fully mature cell plate in the presence of a spreading force (Figure  3B). Within the theory and the variational approach, we ﬁnd that this is possible if the spontaneous curvature decreases to a threshold value (∼0.015 $nm^{-1}$) with larger cell plate area, that is, in the presence of a spreading force. From an energy perspective, this suggests that TVNs, TNs, as well as fenestrated sheets, should be unstable as compared to a single oblate spheroid, and thus transition of their morphology to one without tubes or fenestrations. While a ﬁnite spontaneous curvature is necessary to explain the origin of stability of the incoming vesicles (Jung et al., 2001), a change in the spontaneous curvature of the membrane is predictable due to the expected changes in membrane composition and protein activity that occurs during cell plate development (McMahon and Boucrot, 2015).

Figure  3B shows the relative instability of selected tubular and fenestrated structures compared to a single oblate spheroid in the presence of a spreading force and zero spontaneous curvature. Less tubular structures are now energetically favorable than highly tubular or fenestrated structures, with a single, complete structure being the most favorable. For structures without fenestrations or gaps (such as 2 × 1 × 0 or 3 × 2 × 0), we can also map a path to a single oblate spheroid if we relax the parameter restrictions that were initially imposed during the variational calculation. Figure  3B shows results with the parameter restrictions in place. Supplemental Figure S2 shows data for a fenestrated structure in the absence (Supplemental Figure S2A) and the presence (Supplemental Figure S2B) of a spreading/stabilizing force, leading to gap shrinkage with the parameter restrictions in place.

To better represent a biological system, we compared an ensemble of 2 × 1 × 0 structures (approximating accumulated fused vesicles forming a network) to a single oblate spheroid of the same combined area. Similar to our earlier calculations, we ﬁnd that in the absence of a spreading force, a single oblate spheroid is less stable, as shown in Figure  3C. The relative instability is magniﬁed with the increase of area, and with the increase in the number of tubular structures. In the presence of a spreading force and a decreased spontaneous curvature, as in Figure  3D, the inverse is true, favoring fewer complex structures. When comparing multiple structures to a single mature structure of the same area, there is no need to enforce the decrease in spontaneous curvature. However, for consistency, results with a zero spontaneous curvature in the presence of a spreading force, and a ﬁnite spontaneous curvature in the absence of a spreading force are shown.

Our simulations also showed that a stiffer membrane, for example, one that is represented by a larger bending modulus, requires a stronger spreading force in addition to a higher pressure difference to transition to a mature cell plate structure as shown in Supplemental Figures S3–S7. However, regardless of the choice of the bending modulus that may arise in different regions of the cell plate due to varying thickness and rigidity, a spreading force is necessary (see Supplemental Figures S3–S7). We also show calculations for larger, highly tubulated fenestrated structures (i.e.10 × 13 ×  4) in Supplemental Figures S8 and S9, which are stable in the absence of a spreading force, but unstable compared to a single oblate spheroid in the presence of a spreading force, further supporting our model. In Supplemental Figures S10 and S11, we animate the 3D evolution and transition of cell plate structures in the presence and the absence of a spreading force as predicted by our model.

### Exploring polysaccharide deposition as a contributing factor to cell plate maturation and model prediction

There are several potential contributing factors during cell plate maturation including cell wall polysaccharides. Given that the model examines the specific transition between TN to a fenestrated sheet and a mature cell plate, the timing of the contributing sources at the lagging zone is critical. Among the different polysaccharides we first examined callose.

**Figure 4:**
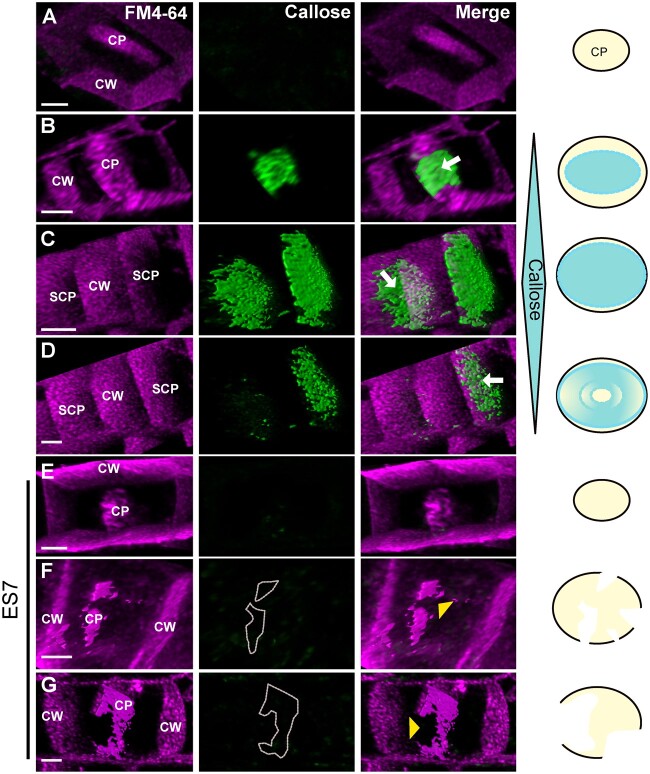
Progression of the cell plate in the presence and absence of callose. A–D, Cell plate progression in the presence of callose. A, It shows an early stage cell plate before the accumulation of callose, while (B–D) represent later cell plate stages including SCP as indicated in Figure  1D. FM4–64 staining (magenta) is used to stain plasma membrane and the cell plate, while Aniline Blue fluorochrome (green) staining shows callose accumulation. Note the transient accumulation of callose in later stages leading to the maturation of cell plate during normal cytokinesis (B–D). C and D represent two snapshots of a time series. C, Two cell plates can be observed, and as maturation continues to D, callose is eliminated from one cell plate indicating its transient nature. Arrows indicate callose accumulation at the cell plate. E–G, Progression of cytokinesis under ES7 treatment for 2 h that inhibits callose deposition. Note that early cell plate development is not affected with ES7 treatment as shown in earlier studies (Park et al., 2014) (E). However, in late stages of cell plate development under ES7 treatment, the absence of callose prevents the transition into a stable mature single structure, leading to characteristic “cell plate stubs” (F and G). CP indicates cell plate, SCP indicates SCP as depicted in Figure  1. CW indicates cell wall. Yellow arrowheads denote lack of callose at cell plate breakage points. Dotted lines in F, G outline the position where callose should be deposited. Images are 3D reconstructions from Z-stacks of live confocal imaging and show single timepoints. C and D are snapshots of a time series. Figures are representative of root tips from a minimum of 10 Arabidopsis seedlings. A schematic representation on the right indicates the accumulation of callose in relation to cell plate development. White gaps at the bottom indicate cell plate fragmentation. Bars = 3 µm.

Live staining of callose in dividing *Arabidopsis thaliana* roots showed a prominent and transient accumulation of the polysaccharide at the lagging zone, starting from the center (Figure  4, B–D). Treatment with Endosidin7 (ES7), which inhibits cytokinetic callose deposition (Park et al., 2014), caused failure of the cell plate to mature into a cross wall. Figure  4, E–G shows an example of arrested cell plate development in the absence of callose, in contrast to normal gradual cell plate maturation concomitant with callose deposition as in Figure  4, A–D. This is consistent with the model’s prediction in the absence of a spreading force, where planar fenestrated and tubular structures accumulate, but do not mature into a stable cross-wall like structure.

**Figure 5:**
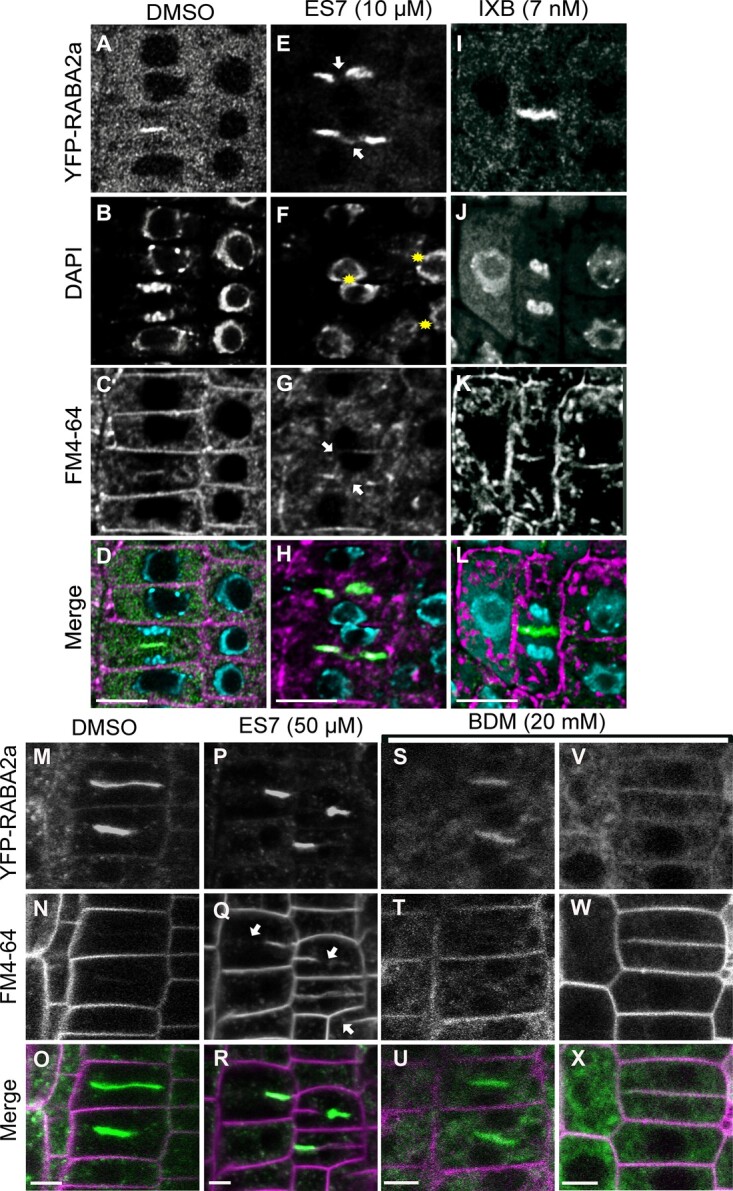
Comparison of chemical inhibitors of cellulose, callose and myosin on cytokinesis. A–L, Evaluation of cytokinesis inhibition under 5 day chemical treatment in Arabidopsis root tips. Under control DMSO treatment normal cytokinesis is observed (A–D). Under ES7 treatment typical cytokinesis defects are observed with the cytokinesis marker RABA2a (E), multinucleate cells (F) are shown by DAPI staining. Under IXB treatment cell plate progression was observed (I) without discernable cytokinetic defects in the form of binucleate cells (J) or cell plate stubs (I, K, and L). Please note cell swelling under IXB treatment. The cytokinesis marker RABA2a is shown in green, while FM4–64 staining of plasma membrane is shown in magenta. Nuclei staining by DAPI are indicated in blue. Samples were stained with FM4–64FX, fixed and stained post fixation for DAPI. Results were observed in at least six roots for each drug treatment. Samples are single scans of fixed cells. Bars = 10 µm. M–X, Effect of 2 h short-term (50 µM) ES7 and the putative myosin inhibitor 2,3-butanedione 2-monoxime (20 mM BDM) treatment in cytokinesis. Under DMSO control treatment normal progression of cytokinesis is observed (M–O). Under ES7 treatment, characteristic cell plate stubs were observed with RABA2a and the plasma membrane stain FM4–64 (P–R). Under BDM treatment, a reduction of RABA2a signal was observed with increase in cytoplasmic pattern (S–X). The cytokinesis marker RABA2a is shown in green, while FM4–64 staining of plasma membrane is shown in magenta. Samples are single scans of live cell confocal imaging. Results were observed in at least six roots for each drug treatment. Bars = 5 µm.

Given the loadbearing role of cellulose, we then examined the effect of cellulose compared to callose inhibition in our experimental conditions. Cellulose inhibition by isoxaben (IXB) treatment led to strong reduction of root growth (Scheible et al., 2001; Worden et al., 2015) and a root swollen phenotype compared to ES7 (Supplemental Figure S12). However, while cytokinesis defects in the form of cell plate stubs, were observed with ES7 treatment (Figure  5, E and Supplemental Figure S12, E and J), this effect was not detectable in IXB treatment (Figure  5 and Supplemental Figure S12, G– J). ES7 treatment caused binucleate cells as a result of failed cytokinesis (Figure  5F); however, this phenotype was not pronounced in the IXB treatment (Figure  5J).

We then included a treatment with the myosin inhibitor 2,3-butanedione monoxime (BDM), interfering with actin-based organelle transport (Samaj et al., 2000; Tominaga et al., 2000; Higaki et al., 2008). Unlike ES7 (Figure  5, P–R), a 20-mM BDM treatment for 2 h led to inhibition of RABA2a trafficking Figure  5, S–X, but not cell plate fragmentation, showing an effect on endomembrane trafficking, that in turn could impact cell plate development.

### Estimating the required polysaccharide synthesis rates for a stabilizing/spreading force

We note that the spreading/stabilizing force can be described by the mean square end-to-end excursion within the Flory self-avoiding polymer theory in two-dimensions (Schulmann et al., 2013). If we assume reasonable values of polymer persistence length and areal density, we require a rate of polysaccharide synthesis close to $\frac{dN}{dt}\;∼\;\;1.75\;\times \;10^{6}\;s^{-1}$ to obtain the spreading force parameter of $\lambda \;=\;\;4\frac{pN}{\;\mathrm{nm}}$. The modeled d*N*/d*t* is biologically achievable given an estimated cellular value of $1.8\;\;\times \;10^{5}\cdot \;s^{-1}$ based on in vitro callose synthase activity (Him et al., 2001) and an average protein concentration in eukaryotic cells (Albe et al., 1990; Milo, 2013). However, in-vitro experimental systems of the relevant polymer(s) synthase(s) in artificial vesicles are required to test this hypothesis. A full description of this derivation is given in the supplemental information corresponding to Supplemental Equations S12–S17.

## Discussion

Although several proteins have been identified that regulate cell plate formation (McMichael and Bednarek, 2013; Smertenko et al., 2017, Gu and Rasmussen, 2021), the mechanisms underlying the complex transition from a vesicle membrane network (TN) to a fenestrated sheet and a mature cell plate are not well understood.

To circumvent these hurdles and to better dissect cell plate maturation, we used biophysical modeling. We developed a model based on the Helfrich free energy for the cell plate surface with the incorporation of a spreading/stabilizing force as an “areal pressure” (force per unit distance). From an energy minimization analysis, we have shown that a planar spreading/stabilizing force is vital for cell plate to transition from vesicle membrane network to a fenestrated sheet and late stage/mature cell plate. We also show that in the absence of a spreading/stabilizing force, the addition of membrane material yields stable TN structures, but that those structures are unable to mature beyond this stage. As shown by different simulations, the need for this spreading/stabilizing force is magniﬁed when we compare a single mature cell plate to multiple smaller vesicle network structures of the same total area. We do not have the detailed molecular scale mechanisms behind the spreading/stabilizing force, but we show that a simple model based upon the expansion of a quasi-two-dimensional self-avoiding polymer captures the correct form (Supplemental Figure S13).

To reach a mature cell plate, our model requires the late-stage onset of the spreading force coupled with a concurrent loss of spontaneous curvature. This raises the intriguing possibility of a common origin to the decrease in spontaneous curvature and onset of a spreading/stabilizing force. In the model, the spreading force is relevant when there is sufficient connection of individual oblate spheroidal vesicles, and it is at this stage that we shut off the spontaneous curvature. The nanoscale surface topography can potentially serve as a direct biochemical signal to activate this process (Lou et al., 2019). The possible tethering of polysaccharides or glycoproteins to the membrane could concomitantly induce spreading and reduce spontaneous curvature by modifying the membrane mechanics. Notably, inhibition of long chain fatty acid affects cell plate maturation (Bach et al., 2011). It is plausible that membrane microdomains control both spontaneous curvature and the onset of a spreading force at the cell plate.

Curvature-stabilizing proteins are active at the cross-sections of tubules and sheet edges of endoplasmic reticulum (Shemesh et al., 2014; Schweitzer et al., 2015). While force generating proteins are involved in the tubulation and membrane material recycling processes (Otegui et al., 2001; Ahn et al., 2017), no proteins have been identified with properties of membrane expansion at the cell plate.

The phragmoplast-driven vesicle delivery is a dynamic and complex process (Buschmann and Müller, 2019) that with the aid of motor proteins can be considered as a spreading/stabilizing force during cell plate maturation. For example, Myosin VIII plays a role in guiding phragmoplast expansion (Wu and Bezanilla, 2014), while several kinesins are involved in the functional organization of the phragmoplast (Buschmann and Müller, 2019). Microtubule directed vesicle delivery occurs at the leading edge; however, it is followed by microtubule depolymerization at the lagging zone, which is the transitional stage that the model describes (Lee and Liu, 2013). Furthermore, inhibition of myosin causes a broader effect on cell plate expansion, as it is involved in general vesicle delivery (Figure  5). Therefore, it is challenging to assign a specific function of motor proteins to cell plate maturation at the lagging zone. Time-lapse experiments directed at the role of motor proteins at the lagging zone will shed light on their contribution to the stabilizing and spreading force that the model predicts.

It is plausible that polysaccharide deposition serves as this stabilizing and spreading role. The matrix polysaccharides hemicellulose and pectin are synthesized in the Golgi apparatus and delivered via vesicles from the beginning of cytokinesis (Moore and Staehelin, 1988; Toyooka et al., 2009; Rybak et al., 2014). Thus, these classes of polysaccharides are unlikely the major players as they do not overlap with the predicted onset of the spreading/stabilizing force, although experimental verification awaits. Callose and cellulose are synthesized directly at the plasma membrane and are excellent candidates for exploration. Our data showed that pharmacological inhibition of cellulose at the root tip inhibited cell elongation in general, while inhibition of callose deposition led to cytokinesis defects consistent with the conformations predicted by the model in the absence of a spreading force. Callose accumulation peaks at the intermediate TN stage, a transitional stage that coincides with loss of membrane volume (Samuels et al., 1995; Segui-Simarro et al., 2004). The timing of callose deposition in late stages when the overall cell plate membrane network “flattens” (Samuels et al., 1995; Segui-Simarro et al., 2004) is consistent with the need of callose in providing a lateral spreading/stabilizing force. Furthermore, the predicted required values of callose deposition are within biological thresholds (Him et al., 2001; Pelosi et al., 2003). Notably, a study by Thiele et al. (2009) indicates that callose is required to establish the connection between the nascent cross-wall and the parental cell wall, rather than stabilizing the young cell plate (Thiele et al., 2009), so that further analysis on the role of callose in the proposed model awaits verification. It is plausible that callose could serve as a scaffold into which other more permanent polysaccharides and proteins are later deposited (Stone and Clarke, 1992; Him et al., 2001). Potential transient interaction with cellulose or other glucans (Miart et al., 2014; Gu et al., 2016; Abou-Saleh et al., 2018) can contribute to a composite that supports the stability of the cell plate and helps the attachment to the parental cell wall. Structural glycoproteins such as extensins (Cannon et al., 2008) can provide a scaffold for polysaccharide deposition, and these altogether can generate the desired spreading/stabilizing force proposed by the model. Further (challenging) experiments are necessary to determine how the possible conformations of different polysaccharides and proteins or their combinations, synthesized in vitro in an artificial membrane setup, can contribute to different magnitudes of spreading/stabilizing force in lipid vesicle networks.

A unique element in the study was the approximation of cellular compartments with testable shapes such as vesicles and complete cell plates with oblate spheroids, fused vesicles and tubular structures with elliptical hyberboloids and their combination in a network. Approximating vesicles, tubulations and their networks in the current model has the potential of a wider application and can be adopted during quantitative assessment of membrane dynamics. It can be used as a basis for addressing the equilibrium of vesiculation (oblate spheroids) and tubulation (elliptic hyperboloids) and applied to ER-intermediate compartments, Golgi, and endosomes in all eukaryotic cells.

In conclusion, our model provides a framework for understanding how membrane structures evolve in the presence of a spreading/stabilizing force and will likely shed light in such transitions that occur beyond cytokinesis.

## Materials and methods

A full description of our model development, parameter set up, plant growth, chemical treatment, and analysis is presented in the supplemental material.

## Supplemental data 

**Supplemental Text S1.**

**Supplemental Figure S1.** Parameters visualized on a representative 2 × 1 × 0 structure.

**Supplemental Figure S2.** Effect of a spreading force visualized in a 8 × 10 × 3 conformation.

**Supplemental Figure S3.** Evolution of single oblate spheroid parameters in the presence of a spreading force.

**Supplemental Figure S4.** Stability tests of various configurations under different bending modulus in the absence of a spreading force.

**Supplemental Figure S5.** Stability tests of various configurations under different bending modulus in the presence of a spreading force and with zero spontaneous curvature.

**Supplemental Figure S6.** Stability tests of multiple 2 × 1 × 0 structures as compared to a single oblate spheroid in the absence of a spreading force and with finite spontaneous curvature.

**Supplemental Figure S7.** Stability tests of multiple 2 × 1 × 0 structures as compared to a single oblate spheroid in the presence of a spreading force and with zero spontaneous curvature.

**Supplemental Figure S8.** Stability tests of tubular/fenestrated structures as compared to a single oblate spheroid in the absence of a spreading force.

**Supplemental Figure S9.** Stability tests of tubular/fenestrated structures as compared to a single oblate spheroid in the presence of a spreading force.

**Supplemental Figure S10.** Evolution/transition of a cell plate structure in the absence of a spreading force as predicted by the model.

**Supplemental Figure S11.** Evolution/transition of a final cell plate structure from Figure S10 in the presence of a spreading force as predicted by the model.

**Supplemental Figure S12.** Effect of IXB and ES7 on cellular organization and root growth.

**Supplemental Figure S13.** Proposed model of polymer deposition generating a two-dimensional spreading force.

**Supplemental Video S1.** Evolution/transition of a cell plate structure in the absence of a spreading force as predicted by the model.

**Supplemental Video S2.** Evolution/transition of a final cell plate structure from Figure S10 in the presence of a spreading force as predicted by the model.

## Supplementary Material

kiab552_Supplementary_Data

## Notes

D.C. and G.D. designed and analyzed research. M.Z.J. did computational work and R.S. did experimental work. M.Z.J. wrote the article, with significant revisions from D.C., G.D. and input from R.S. and V.B. R.S. contributed to the “Materials and methods”, the experimental data presented and the relevant analysis and interpretation, as well as figure illustrations and captions. All authors read and approved the final version of the manuscript.

The authors responsible for distribution of materials integral to the findings presented in this article in accordance with the policy described in the Instructions for Authors (https://academic.oup.com/plphys/pages/general-instructions) are G. Drakakaki (gdrakakaki@ucdavis.edu) and D.L. Cox (cox@physics.ucdavis.edu).
