# Supplementary material for: A biophysical model for plant cell plate maturation based on the contribution of a spreading force
Source: Plant Physiol. 2021 Nov 27;188(2):795–806. doi: 10.1093/plphys/kiab552 (PMC8825336; doi:10.1093/plphys/kiab552)
Supplement: kiab552_Supplementary_Data [file kiab552_supplementary_data.zip › Supplemental_Material_and_Figures.pdf]

Supplementary Information

A Biophysical Model for Plant Cell Plate Maturation Based on the Contribution of a Spreading Force

M.Z. Jawaid, R. Sinclair, V. Bulone, D.L. Cox[1], G. Drakakaki[1]

[1]Senior authors

This PDF file includes:

Supplemental text
Supplemental Figures S1 to S13

Supplemental Text

Supplemental Material and Methods

Parameter Set Up

To implement energy minimization on a parameterized basis set defined at a particular surface area, we first identified the parameter space for a given conformation that yielded the desired area. For a single oblate spheroid, only two parameters are needed, namely, the radius along the major axis, 'a', and the radius along the minor axis, 'c'. However, for the consolidated tubular networks and emerging fenestrated structures, one must account for continuity between the oblate spheroid and the elliptic hyperboloid, while also making the relevant corrections in area. This continuity can be achieved by matching the slopes of the elliptical hyperboloid with the oblate spheroid along the primary axes, while enforcing contact.

A normal one-sheeted elliptical hyperboloid centered at a distance d along the x axis can be described by the following equation:

$$\frac{y^2}{a_h^2} + \frac{z^2}{b_h^2} - \frac{(x-d)^2}{c_h^2} = 1 \qquad (S1)$$

Here, a_h is the skirt radius along the xy-plane, b_h is the skirt radius on the xz-plane, and c_h describes the elongation along the y-axis, where the xy-plane defines the equatorial plane, and the z-axis is the polar axis. Supplemental Fig. S1 shows these parameters for a hyperboloid in a 2x1x0 structure.

By enforcing continuity with an oblate spheroid at the origin, we can derive the following relationships for d and b_h such that:

$$d = \frac{1}{2}\sqrt{a^2 - a_h^2}\sqrt{a^2 + c_h^2} \qquad (S2)$$

$$b_h = a_h \frac{c}{a} \qquad (S3)$$

Using this, the length of the hyperboloid l is given by:

$$l = 2d\left(1 - \frac{a_h^2}{a_h^2 + c_h^2}\right) \qquad (S4)$$

Using these restrictions, we can form connected structures of oblate spheroids and hyperboloids to form approximate representations of tubular and fenestrated structures. Additional demands that are placed by specific conformations such as in complex emerging fenestrated structures (for example 6x9x3) were taken into account by eliminating choices of hyperboloids that would cause clashes.

Thus, our complete parameter space for a given conformation at a particular area is given by a list of values of (a, c, a_h, l), where the corresponding area is calculated by numerical integration methods up to an error tolerance of 0.01%, taking into account spatial constraints.

Energy Minimization

For our energy minimization calculations, we treat the membrane boundary of the cell plate as an incompressible two-dimensional surface to a first approximation, with certain defined characteristics. In reality, each lipid bilayer has a finite thickness of 4-6 nm (Rawicz et al., 2000), however, it is justified to treat the surface of the cell plate as two-dimensional due to the smallness of its thickness compared to the sizes of the overall structures in the cell plate. It can also be shown that lipid bilayers present a high level of incompressibility due to the energy penalty associated with areal stretching being significantly higher compared to membrane deformations due to bending (Lazaro et al., 2015). This is similar to the approach taken by Choksi et al. (Choksi et al., 2013) and Sarasij et al. (Sarasij et al., 2007).

At a specified area, we define a free energy of our membrane surface that is essentially the Helfrich energy (Helfrich, 1973) with the addition of a novel term to model the spreading force, possibly due to polysaccharide deposition, as given in Eq. (1). We discuss the possible origin of this spreading force within a simple model elsewhere. Finally, we consider the surface area of the cell plate as a proxy for cell plate development stage/time and then we minimize this energy for a given surface area.

Modified Helfrich Energy

The modified Helfrich energy (Helfrich, 1973) is described as follows:

$$E = E_{bending} + E_{pressure} + E_{tension} + E_{gaussian} + E_{spreading} \quad (1)$$

The first term describes the bending energy over the closed membrane surface(s) of the cell plate. It is given by:

$$E_{bend} = \frac{K_B}{2} \oint (H_1 + H_2 - c_o)^2 \, dA \quad (S5)$$

Where K_B is the bending modulus for the membrane surface, H_1 and H_2 are the principal curvatures at a point on the surface, and c_o is the spontaneous curvature, or the preferred curvature for the membrane. For simplicity, we assume that the bending modulus is time independent. We allow for the spontaneous curvature, c_o, to be time dependent, which reflects potential differences in membrane composition during cell plate evolution.

The next term is the pressure energy which results from the difference in osmotic pressure between the inside and the outside of the cell plate, such that $\Delta p = p_{out} - p_{in}$. It is given by:

$$E_{pressure} = \int \Delta p \, dV \quad (S6)$$

The third term is the energy associated with the surface tension of the membrane, given by:

$$E_{tension} = \oint \gamma \, dA \quad (S7)$$

We assume that the surface tension given by γ is a constant. Since we assume that our system locally equilibrates in time at a constant area, this term only adds a constant energy equal to the area at that time, multiplied with the surface tension.

The fourth term is the Gaussian bending energy term, given by:

$$E_{gaussian} = 4\pi K_G(1-g) \quad (S8)$$

Here, K_G is the Gaussian bending modulus, and g is the genus of the surface. This is a result of the Gauss-Bonnet theorem (Lee, 1997). Finally, we consider the surface area of the cell plate as a proxy for cell plate development time and then we minimize this energy for a given surface area.

Finally, we introduce the novel element of a spreading force, which is analogous to a two-dimensional pressure that acts against the periphery of the cell plate structure along the equatorial plane. In this interpretation, λ has units of force/length, with the energy representation as follows:

$$E_{spreading} = -\int_{plane} \lambda \, dA \quad (S9)$$

It is important to note that the integral in Eq. (S9) is over the equatorial plane of closed cell plate surfaces. Therefore, fenestrations are not integrated over.

As with the spontaneous curvature, we allow for the spreading force coefficient λ to be time dependent, representing, e.g., the "turning on" of callose production in an expanding plate.

We use established values for the bending modulus K_B (Dimova, 2014), as well as the Gaussian bending modulus K_G (Hu et al., 2012), and the surface tension γ (Fischer-Friedrich et al., 2014). However, the pressure difference Δp was phenomenologically determined. The bending modulus is sensitive to the environment of the cell as well as the membrane type and conformation (Dimova, 2014), and therefore there is a range of available literature values. We decided to test over the full range of literature values ranging from about 62.5pNnm to 200pNnm (roughly corresponding to a range of 15k_bT to 50k_bT).

Note that we have excluded a line tension term from Eq. (3). Such a term gives the wrong morphological sequence in time for the plate, and requires unphysically large values to stabilize the mature, genus 0 plate structure.

Calculation of area elements

To calculate the mean curvature and the area elements of the parameterized structures, we employed the following methods:

$$H = \frac{1}{2}(H_1 + H_2) = \frac{eG - 2fF + gE}{2(EG-F)^2} \quad (S10)$$

$$dA = \sqrt{EG - F^2}\,dudv \quad (S11)$$

Where $E, F,$ and G are coefficients of the first fundamental form (line element) and $e.f,$ and g are coefficients of the second fundamental form (shape tensor), and u and v parameterize the surface. The selection of E,F, G are based standard practices as described in (Abbena et al., 2017). Thus, we can calculate the $E_{bending}$ from Eq. (1) for any conformation given the conformation type and its corresponding parameter space.

The code used to calculate the bending energies and the surface areas of the aforementioned conformations are given in the following public github repository:
https://github.com/zaki92/Bending_Energy_Calculations

Plant Growth

Arabidopsis thaliana seedlings of Col-0 were used in this study. Seeds were sterilized using 30% (v/v) sodium chlorate in ethanol (absolute) with 0.06% (v/v) of Triton X-100 (Sigma-Aldrich). Seeds were plated on 0.25 Murashige and Skoog medium (1.15 g L−1 Murashige and Skoog minimal organics salt, 10 g L−1 Suc, 5 g L−1 Phytagel (Sigma-Aldrich), and cold vernalized for 48 h at 4°C in the dark, after which plates were transferred to a plant growth chamber for seedling growth. Plants were grown in temperature- and photoperiod-controlled environments, set to long-day (16-h-light/8-h-dark cycle) conditions, using fluorescent light (at 100 to 150 mmol quanta photosynthetically active radiation (PAR) m^{-2} s^{-1}) at 22 to 24°C.

Chemical treatment and Imaging

Four day old Arabidopsis seedlings were treated with 50 µM Endosidin7 (ES7), 20 mM 2,3-Butanedione monoxime (BDM) and Dimethylsulfoxide (DMSO) in 0.25 MS medium for two hours as previously described (Park et al., 2014; van Oostende-Triplet et al., 2017).
A Leica SP8 or Zeiss 710 confocal microscope was used for imaging. Aniline blue fluorochrome (Biosupplies Australia) was used to detect callose deposition, while the lipophilic membrane dye FM4-64 (10 µM) (ThermoFisher Scientific) was used to stain the plasma membrane and the developing cell plate and DAPI was used for nuclei staining. For DAPI staining FM4-64FX stained seedlings were fixed in 4% PFA (Park et al., 2014) and post fixation was stained with1 µg mL−1 4′,6-diamidino-2-phenylindole (DAPI). 6-10 seedlings were imaged per individual treatment. Fluorescence signals of callose stained by Aniline blue fluorochrome and DAPI (excitation 405 nm, emission 415-500 nm), FM4-64 (excitation 510 nm, emission 620-759), YFP-RABA2a (excitation 510 nm, emission 520-570-) were collected with 40x (water), 63x (oil) objectives. Z stacks were generated across the volume of full cell and were subsequently deconvolved with Huygens (SVI). 3D reconstructions were prepared using Imaris, Bitplane and figures were assembled using Affinity Designer.
Four day- chemical treatments were performed on seedling germinated in 0.25 MS agar media supplemented with the indicated inhibitors:7 or 10 nM isoxaben (IXB), 10 µM ES7 and DMSO.
Root length was quantified using Image J (Schneider et al., 2012) as previously described (Worden et al., 2015). Multi- factor analysis of variance (ANOVA) was performed using R x 64 version 4.0.3 (R Core Team, 2017) in Rstudio (RStudio, PBC), version 1.3.1093 with the basic ANOVA function. Least square means (LS means) analysis was performed using the emmeans package (version 1.5.3) and the multcomp package (version 1.4- 15) in R and the p-value adjusted for multiple comparison using the

Tukey method. The graphs were generated using ggplot2 package (version 3.3.3) and the emmeans package. Letters assigned by LS means, $p = .05$

Supplemental Results

Results with full range of bending moduli

The data in Supplemental Figs. S3-S7 show additional calculations in a range of bending moduli. A bending modulus of 62.5 pN-nm (about 15K_bT) corresponds to the lower range of bending moduli, consistent with published data (Dimova, 2014), while a bending modulus of 200 pN-nm (about 50K_bT) corresponds to the higher range. It is important to note that the key outcome remains the same, a finite spreading force coupled with a decrease in spontaneous curvature is essential for a transition to a single, complete cell plate structure, regardless of the choice of bending modulus.

Fenestrated structures data at higher areas

The supplemental data in Figs. S8-S9 show ΔE_{min} calculations for different types of emerging fenestrated structure conformations at larger cell plate areas. In the absence of a spreading force, and with finite spontaneous curvature, larger, more tubulated fenestrated structures are more stable than a single cell plate. In the presence of a spreading force and with decreased spontaneous curvature, a transition to a single, mature cell plate structure is energetically favorable. Fig. S2 shows the effect of the spreading force when restricted to a parameter basis set for a chosen structure, which in Fig. S2 is 8x10x3. When these parameter restrictions are removed, we see structures change conformation types, as seen in Supplemental Videos S1, S2.

Emergence of Spreading Force from Two-Dimensional Self-Avoiding Polymer Physics

As a finite thickness polymer, the mean square extent of a polysaccharide polymer is subject to the law of self-avoiding polymers, *viz.*

$$< R^2 > = b^2 N^{2\nu} \qquad (S12)$$

with, per Flory theory (Schulmann et al., 2013),

$$\nu = \frac{3}{2+d} \qquad (S13)$$

obtained from a balancing of two entropic effects of self-avoidance and entropic springiness (high probability of zero end-to-end distance). Here b is the size of a polymer link, of order the persistence length. In two dimensions, $\nu = \frac{3}{4}$. Hence, as a potential polymer is deposited forming a network with other polysaccharides or glycoproteins in the lumen of the cell plate and potentially tethers to each membrane side of the inner cell plate, a radial pressure can be exerted at the edge (Supplemental Fig. S10). The radial growth speed v_F is given by

$$v_F = \frac{1}{2\pi R}\frac{d < R^2 >}{dt} = \frac{3}{2}\frac{b^2}{2\pi R} N^{\frac{1}{2}}\left(\frac{dN}{dt}\right) \qquad (S14)$$

and the rate of change of area is

$$\frac{dA}{dt} = \pi \frac{d < R^2 >}{dt} = \frac{3\pi}{2} b^2 N^{\frac{1}{2}} \left(\frac{dN}{dt}\right) \quad (S15)$$

Hence, the magnitude of the radial force acting on the edge of the plate is (m_c is the mass of a constituent monomer, i.e., glucose, and σ_c is the areal density of polymer in the plate)

$$\frac{dp}{dt} = m_c \sigma_c \frac{dA}{dt} v_F = \frac{9\pi}{8} b^4 \frac{m_c \sigma_c N}{\pi R} \left(\frac{dN}{dt}\right)^2 \quad (S16)$$

The spreading force λ or areal pressure is the total work done per unit area in expanding the plate a radial distance dR so

$$\lambda = \frac{d|W|}{dA} = \frac{dp}{dt}\frac{1}{R} = \frac{9\pi}{8} b^4 m_c \sigma_c^2 \left(\frac{dN}{dt}\right)^2 \quad (S17)$$

Hence, in this simple model, the spreading force is directly related to the production rate of a polymer in the cell plate. Interestingly callose deposition with the size of a polymer link $b \sim 200\ nm$ (Him et al., 2001; Pelosi et al., 2003), and a reasonable value for σ_c, provides an estimate of $\frac{dN}{dt} \sim 1.75 \times 10^6\ s^{-1}$ for $\lambda = 4 \frac{pN}{nm}$ which aligns well with the model.

We recognize two important assumptions entering this two-dimensional picture of the spreading force: 1) the only way to generate such a quasi-two-dimensional force from a self-avoiding polymer network is by breaking the symmetry. There is already a symmetry breaking to the nascent cell plate via the phragmoplast guided vesicle delivery which aligns arriving vesicles and vesicle diameter sets the basic cell plate thickness. To prevent a 3D self-avoiding polymer growth, we must assume there is confinement of the growth which necessitates tethering of the polymer network to each side of the new cell wall. 2) Because of the out of equilibrium dynamics of polysaccharide production and the absence of equilibration from the cytosol to the interior of the cell plate, we are approximating the areal pressure at the edge of the cell plate using Newton's second law and relating that to potential polysaccharide production rates. This contrasts the usual energy supplied polymer ratchet model of actin or microtubules which assumes energy supply by ATP or GTP hydrolysis and a steady state of monomers and polymers, with force generation arising from monomers added at the leading edge of the polymer and removed from the trailing end (Mogilner et al., 2003).

Line Tension

We additionally considered the notion of a line tension at the membrane boundary lying on the equatorial plane. The energy of such a line tension would be $E_{spreading} = -\int_{boundary} t\ dl$, where t would have units of force, and dl is the line element along the boundary of a cell plate structure. However, we found that a line tension was unable to reflect the tendency of the spreading force to widen and expand tubular networks and it rather tended to proliferate the length of the membrane boundary instead. This resulted in the increase of the sizes of the fenestrations in fenestrated structures. Attempts to recreate experimentally observed structures of genus zero using this form of the line tension such as those found in Fig. 2E resulted in unrealistic values for t (upwards of 200pN). Additionally, this would be nonzero for a

tangential force at the boundary, where the spreading force is anticipated to be normal to the boundary. We concluded, therefore, that a spreading force that is analogous to a two-dimensional pressure acting at the boundary of the equatorial plane is best represented by Eq.(S9).

Supplemental Figures

A

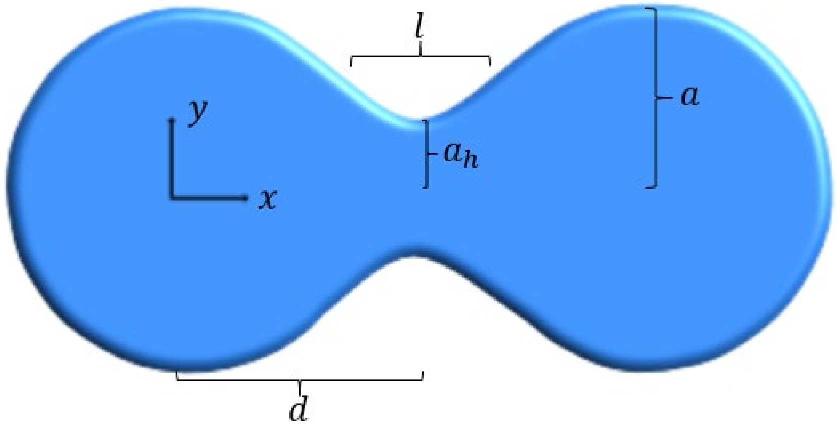

B

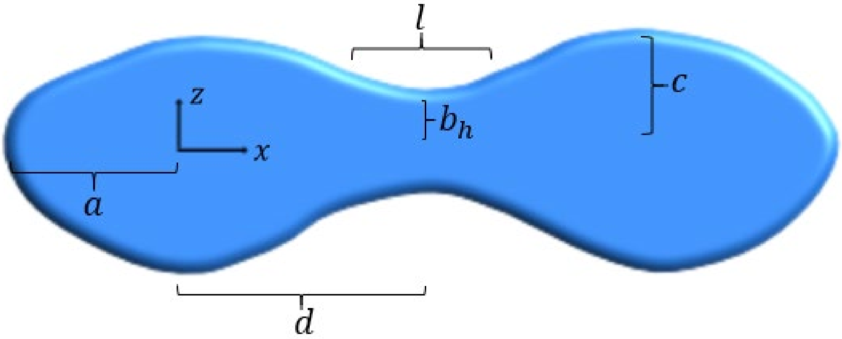

Supplemental Figure S1. Parameters visualized on a representative 2x1x0 structure.
To enforce continuity between an oblate spheroid of given parameters (a, c) and an elliptic hyperboloid with parameters (a_h, b_h, c_h), we can calculate d and b_h are dependent variables, a full conformation can be described by the type of conformation and the parameter set (a, c, a_h, l), or equivalently (a, c, a_h, c_h). The perpendicular arrows show the respective axes of the conformation. **A,** shows the top view of the conformation, while **B,** shows the side view of the same conformation.

A

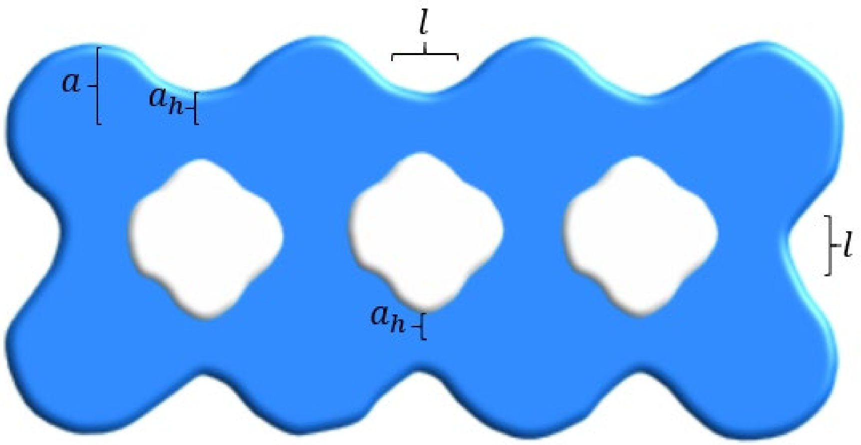

B

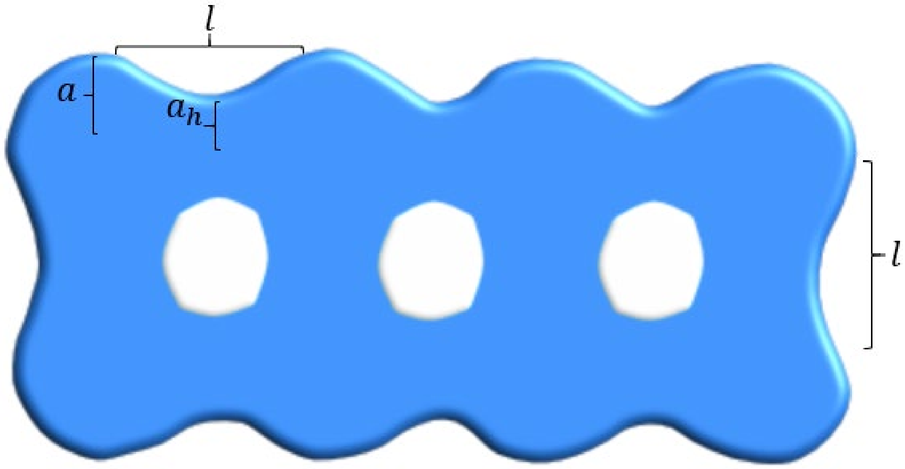

Supplemental Figure S2. Effect of a spreading force visualized in a 8x10x3 conformation.
The parameters shown here were extracted after energy minimization calculations on an 8x10x3 structure with parameter restrictions in place. In the absence of a spreading force, larger fenestrations, and narrower tubular connections are predicted, as shown in a top view in **A**. This structure has an area of 2x10$^5 nm^2$, while the parameters (a, c, a_h, l) are given by (52,31.5,20,25.8)nm. As a spreading force is turned on and the spontaneous curvature is decreased, the tubular connections widen, thereby shrinking the fenestration sizes, as shown in a top view in **B**. For the same area, the parameters change to (58,24,33.5,25.41)nm. If we relax the imposed parameter restrictions in the presence of a spreading force, the resulting structure would reach a single oblate spheroid with $a = 173nm, c = 25nm$.

A

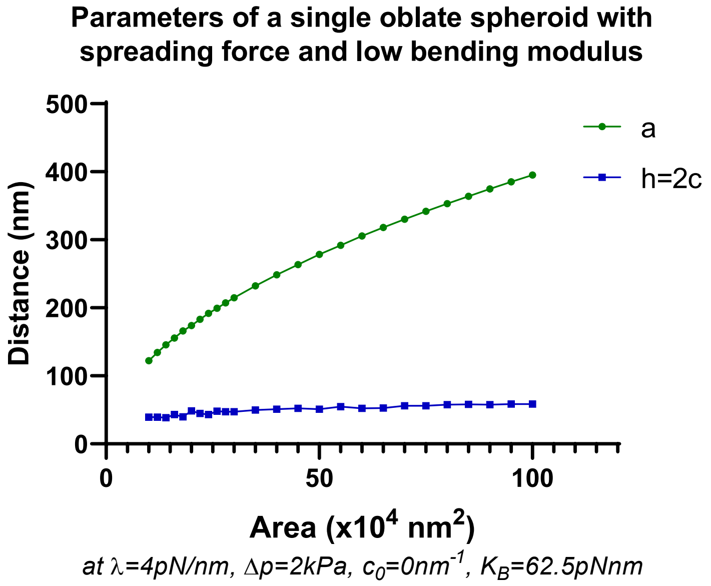

B

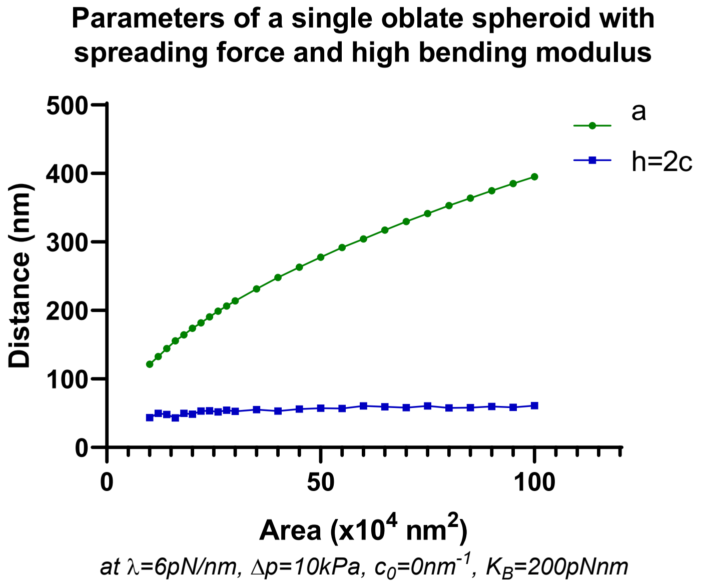

Supplemental Figure S3. Evolution of single oblate spheroid parameters in the presence of a spreading force.
Results with extremal values of the bending modulus are shown in **A** and **B**. Despite the increasing area, the height (h) remains in the 40-80 nm region. With a smaller bending modulus, as in **A**, a smaller value of the spreading force parameter λ and pressure difference Δp is required to maintain the height within the desired region for the specified areas. With a larger bending modulus, as in **B**, larger values of λ and Δp are required.

A

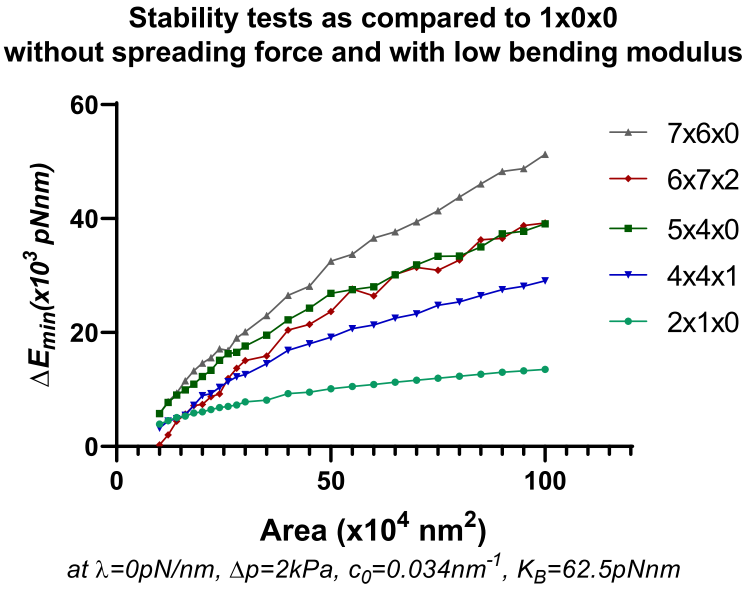

B

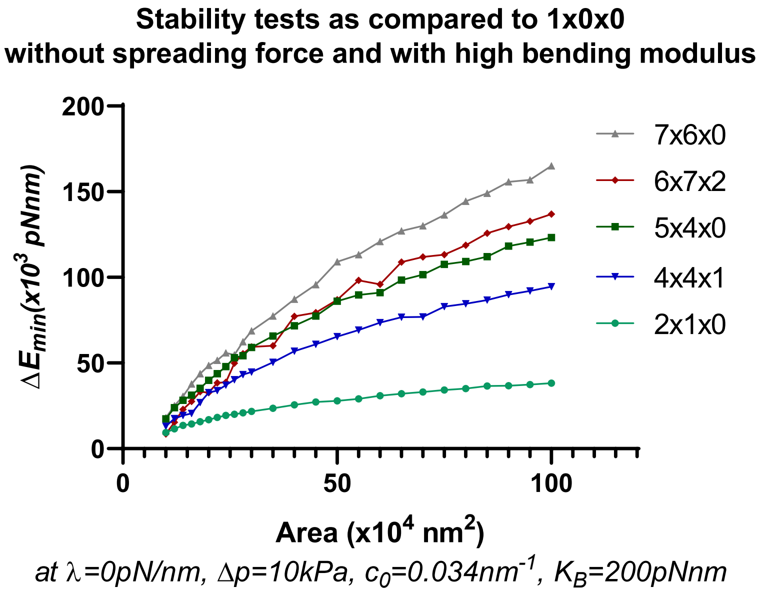

Supplemental Figure S4. Stability tests of various configurations under different bending modulus in the absence of a spreading force.

A, Stability tests for a small value of bending modulus while **B** shows calculations for a larger value of bending modulus. A positive value of ΔE_{min} indicates relative stability of the labelled conformation as compared to a single oblate spheroid. Note that in the absence of a spreading force and finite spontaneous curvature, increasingly tubular and fenestrated structures are more stable as compared to a single oblate spheroid. The different values of $\Delta \mathrm{p}$ and λ arise due to the constraints on structure thickness as shown in Fig. 2 and Supplemental Fig. S3.

A

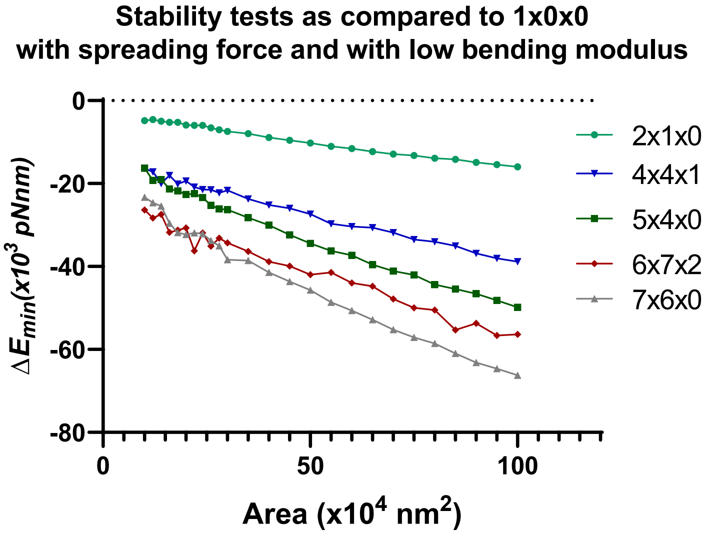

at λ=3pN/nm, Δp=2kPa, c_0=0nm^{-1}, K_B=62.5pNnm

B

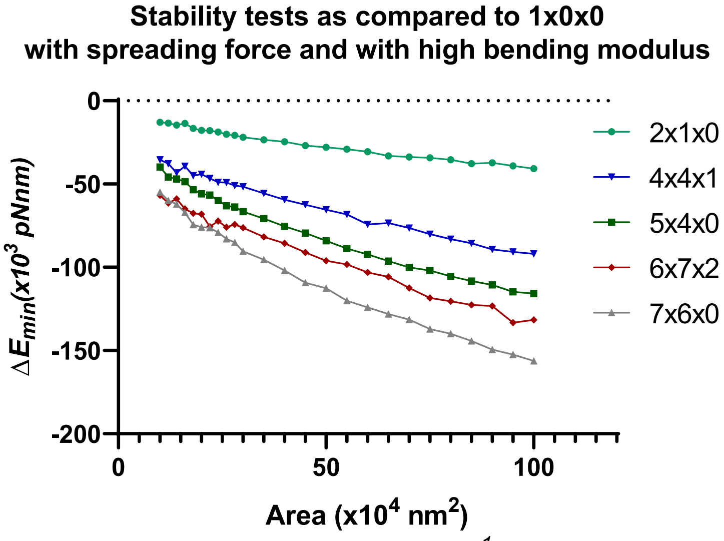

at λ=6pN/nm, Δp=10kPa, c_0=0nm^{-1}, K_B=200pNnm

Supplemental Figure S5. Stability tests of various configurations under different bending modulus in the presence of a spreading force and with zero spontaneous curvature.
A, Stability tests for a lower value of bending modulus and **B** higher value of bending modulus. Note that with the presence of a spreading force and with zero spontaneous curvature, increasingly tubular and fenestrated structures (i.e.7x6x0) are increasingly unstable as compared to a single oblate spheroid, indicating the energetic favorability for cell plate structures to mature to a disk like shape. A positive value of ΔE_{min} indicates relative stability of the labelled conformation as compared to a single oblate spheroid. The different values of Δp and λ arise due to the constraints on structure thickness as shown in Fig. 2 and Supplemental Fig. S3.

A

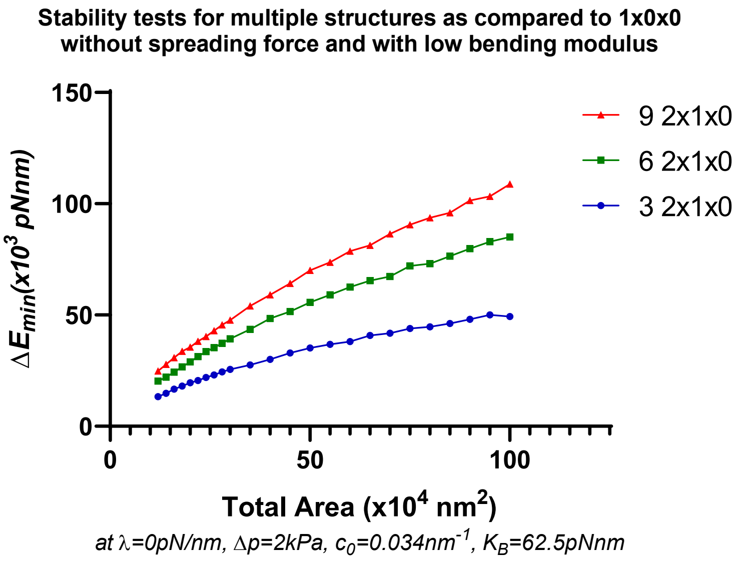

B

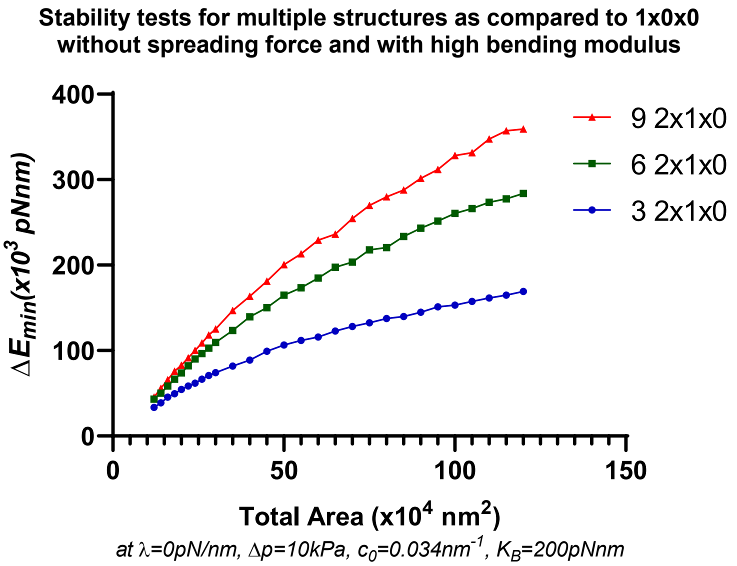

Supplemental Figure S6. Stability tests of multiple 2x1x0 structures as compared to a single oblate spheroid in the absence of a spreading force and with finite spontaneous curvature. ΔE_{min} of multiple 2x1x0 structures as compared to a single oblate spheroid under for a low value of bending modulus **A** and high value of bending modulus **B** are shown. Note that in the absence of a spreading force and with finite spontaneous curvature, tubular structures are energetically favorable in these conditions, thereby modeling a membrane network stage. A positive value of ΔE_{min} indicates relative stability of the labelled conformation as compared to a single oblate spheroid. The different values of $\Delta \mathrm{p}$ and λ arise due to constrains on structure thickness as shown in Fig. 2 and Supplemental Fig. S3.

A

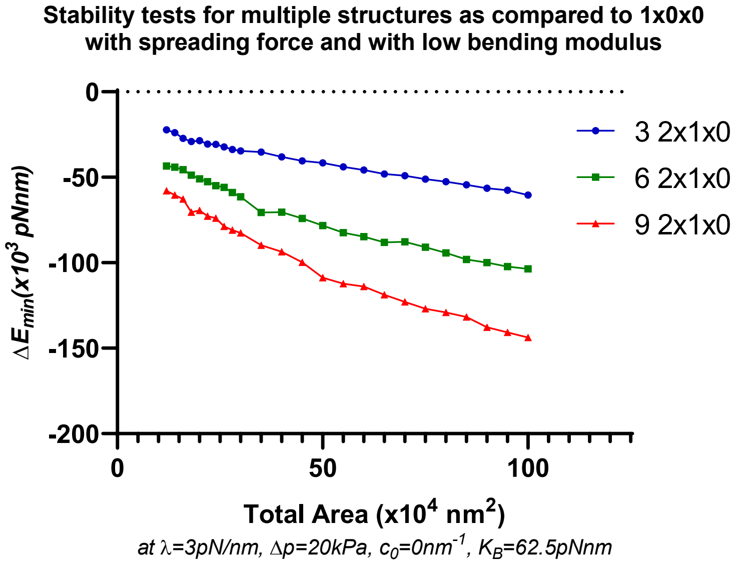

B

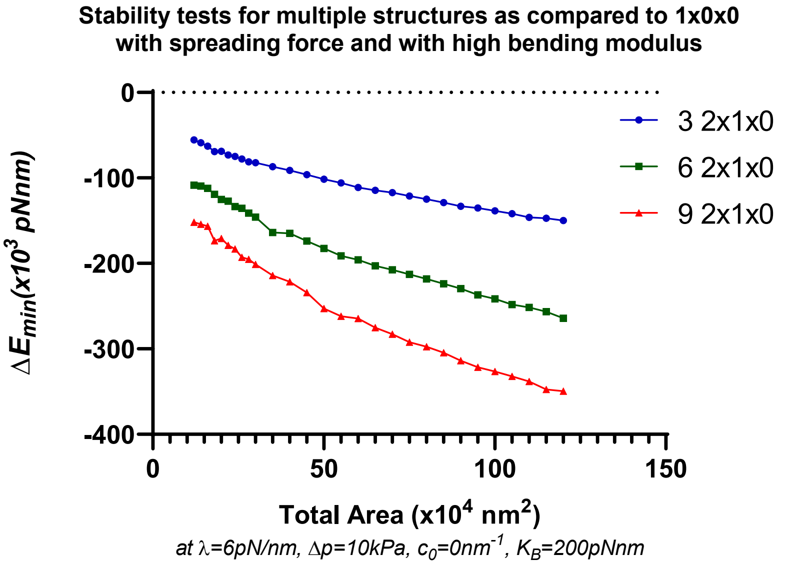

Supplemental Figure S7. Stability tests of multiple 2x1x0 structures as compared to a single oblate spheroid in the presence of a spreading force and with zero spontaneous curvature.
In the presence of a spreading force and with zero spontaneous curvature, tubular structures are unstable compared to a single oblate spheroid, thereby indicating the energetic favorability of structures fusing to form larger, more mature structure(s). **A,** shows results for a small value of bending modulus while **B,** shows results for a larger value of bending modulus. A positive value of ΔE_{min} indicates relative stability of the labelled conformation as compared to a single oblate spheroid. The different values of Δp and λ arise due to the limitations on structure thickness as shown in Fig. 2 and Supplemental Fig. S3.

A

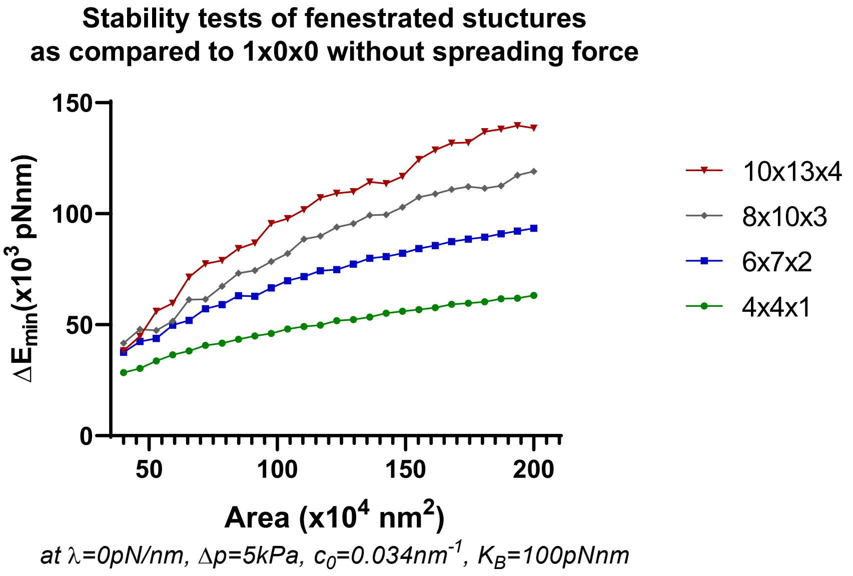

B

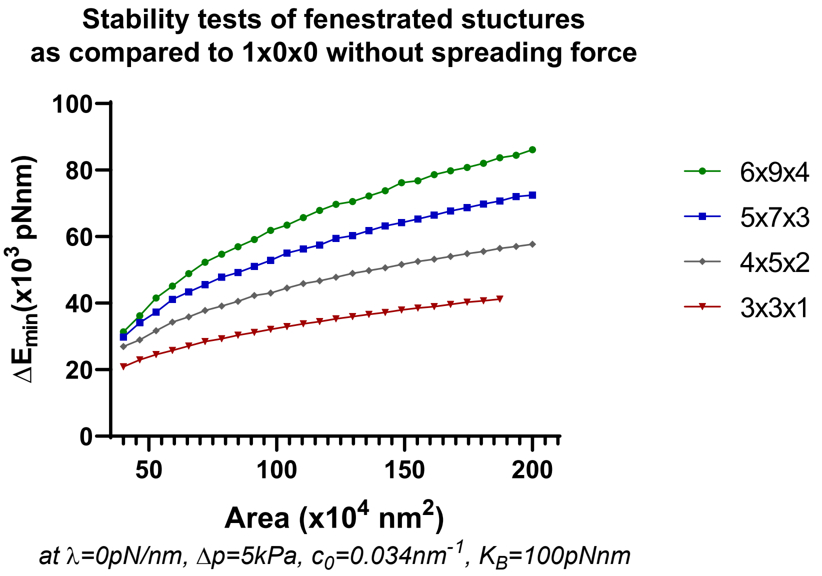

Supplemental Figure S8. Stability tests of tubular/fenestrated structures as compared to a single oblate spheroid in the absence of a spreading force.
In the absence of a spreading force, and with finite spontaneous curvature, fenestrated and tubular structures are, in general, more stable than a single oblate spheroid. This relative stability is magnified with the increase in area particularly for heavily tubular structures (10x13x4 in **A,** 6x9x4 in **B**), consistent with observations at tubular network/very early fenestrated sheet stages.

A

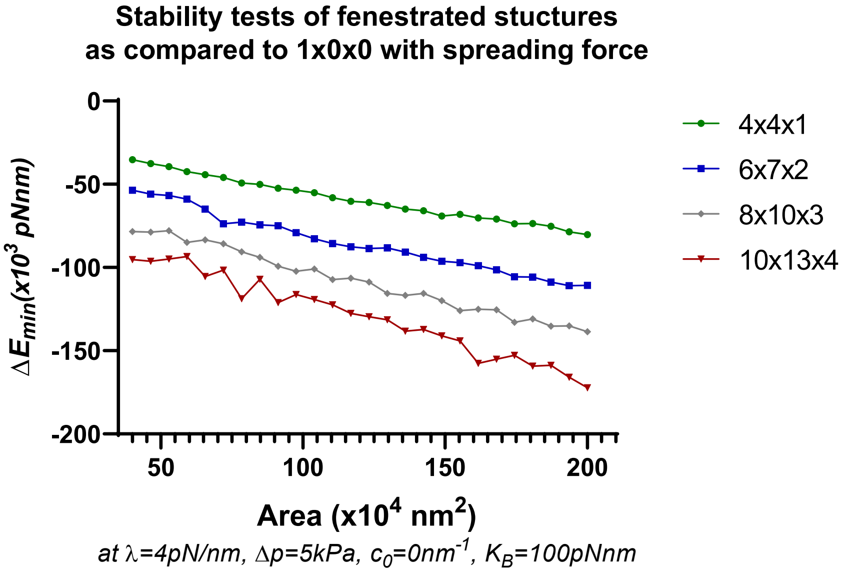

B

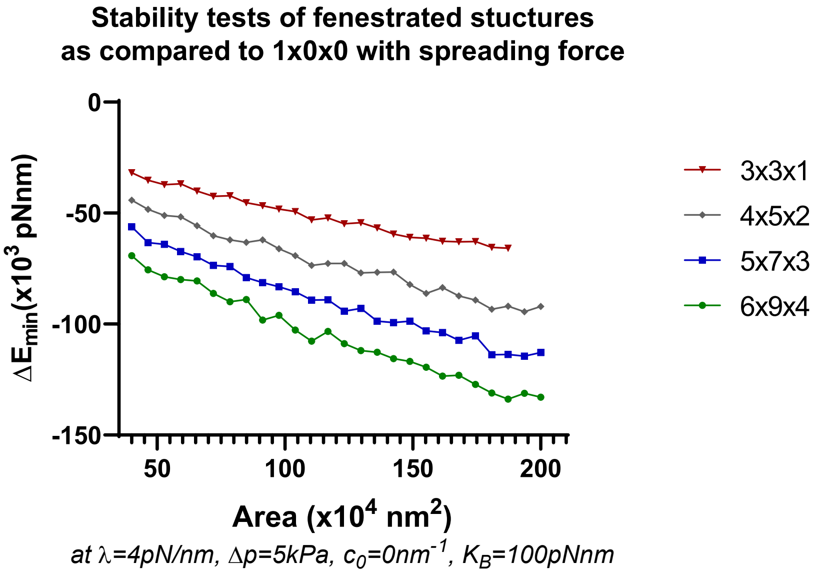

Supplemental Figure S9. Stability tests of tubular/fenestrated structures as compared to a single oblate spheroid in the presence of a spreading force.

A, B, In the presence of a spreading force, and with decreased spontaneous curvature, a single oblate spheroid is more stable compared to larger, tubular, fenestrated structures. This indicates the necessity of a spreading force to incur a transition from a tubular/ fenestrated sheet stage to a single mature cell plate structure.

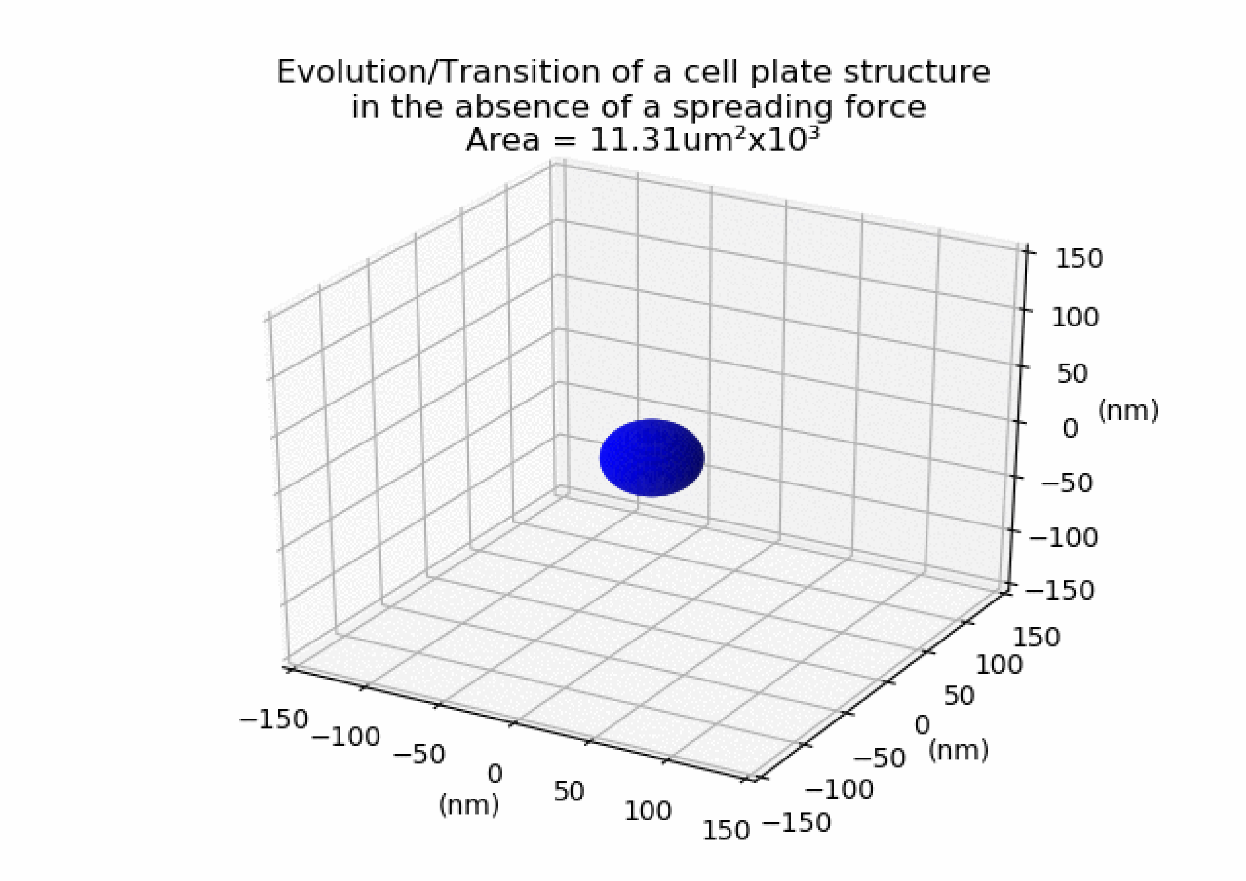

Supplemental Figure S10 and Supplemental Video S1. Evolution/transition of a cell plate structure in the absence of a spreading force as predicted by the model.
Still image from Supplemental Video S1. As membrane area increases in the absence of a spreading force (with the same parameters as in Fig. 3A), a vesicle eventually transitions to an oblate until a tube (shown in red, modeled by elliptical hyperboloids as shown in Fig 2B) forms in between, after which the tubular regions grows longer and narrower, taking away membrane material from the oblate regions (blue, modeled by oblate spheroids as in Fig 2A). Here, we see a transition from a 1x0x0 structure to a 2x1x0 structure in the absence of a spreading force. If the area were to continue increasing in the absence of a spreading force, we would likely see the formation of more tubes (structures like 3x2x0, 4x3x0) as well as fenestrations in some cases (4x4x1, 6x7x2..), as predicted in Fig.3B. Scale (x,y,z) (300nmx300nmx300nm)
For full animation please see supplemental Video S1.

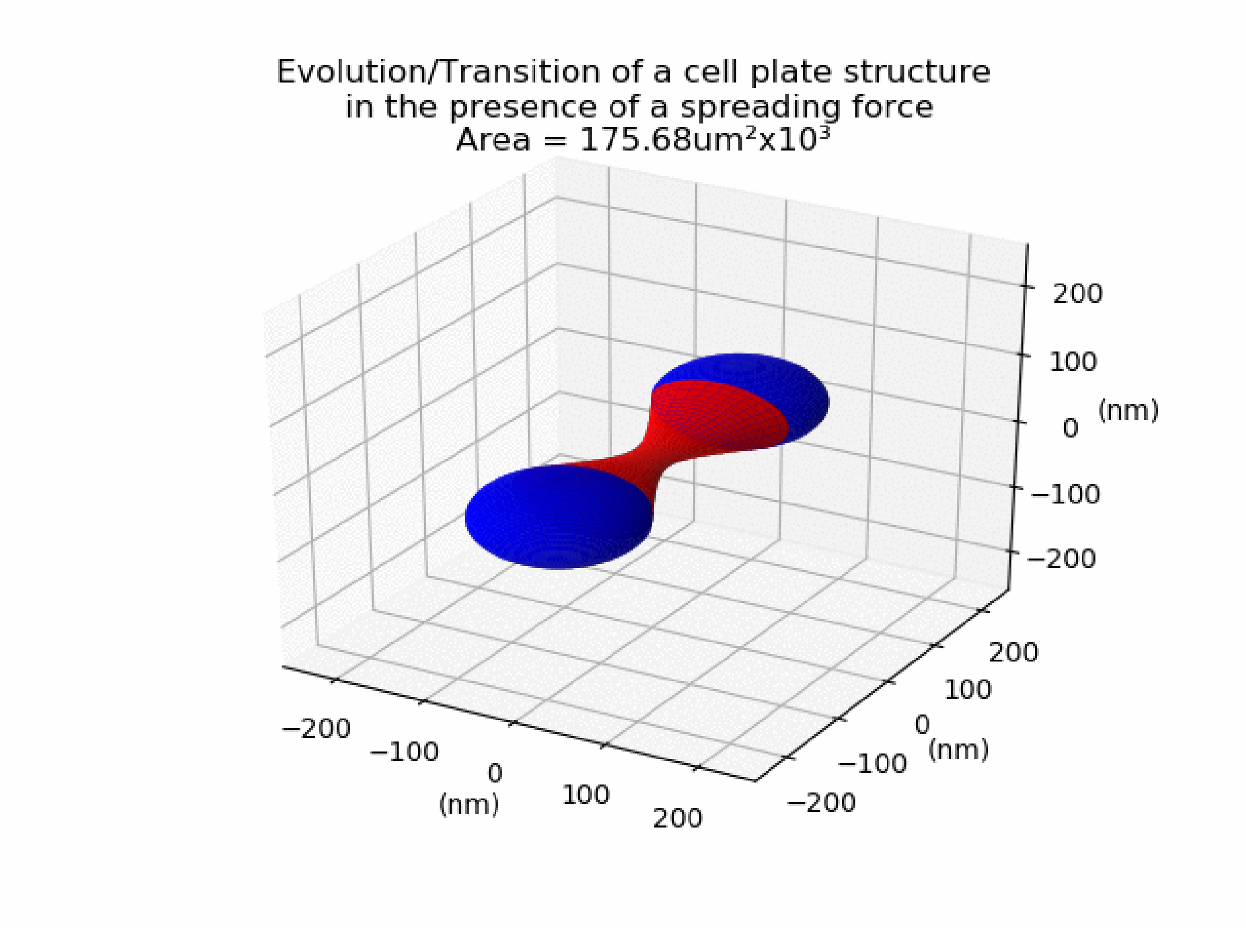

Supplemental Figure S11 and Supplemental Video S2. Evolution/transition of a final cell plate structure from Fig. S10 in the presence of a spreading force as predicted by the model. Still Image from Supplemental Video S2. As membrane area increases in the presence of a spreading force (starting from the shape in Fig. S11, now using the same parameters as in Fig. 3B), the tubular regions (shown in red, modeled by elliptical hyperboloids as shown in Fig 2B) fatten and widen until the structure is fully oblate. Here, we show a transition from a 2x1x0 to a 1x0x0 structure. We note that such a transition is not possible without a planar spreading/stabilizing force. If we were to start from a more tubular or fenestrated configuration with the same area, we would ultimately arrive at the same final shape (1x0x0). Scale (500nmx500nmx500nm). For full animation please see supplemental Video S2.

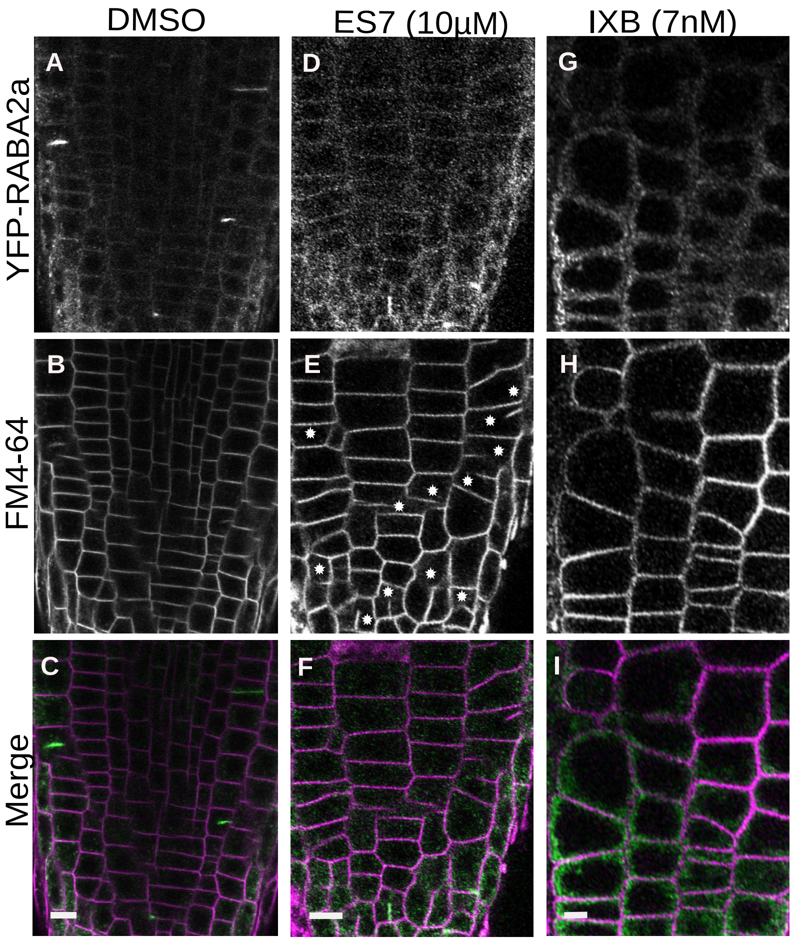

J. Quantif cation of Cytokinesis Defects

Treatment	Normal	Swollen	CW Gaps*	Total
DMSO	228 (100%)	0	0	228
ES7 (10µM)	108 (72.48%)	0	41 (27.5%)	149
IXB (10nM)	86 (85.14%)	15 (14.9%)	0	101

*Note: def ned as cells that contain discontinuous cell walls(CW)or cell plate

K. Root Length in the Presence of Inhibitors

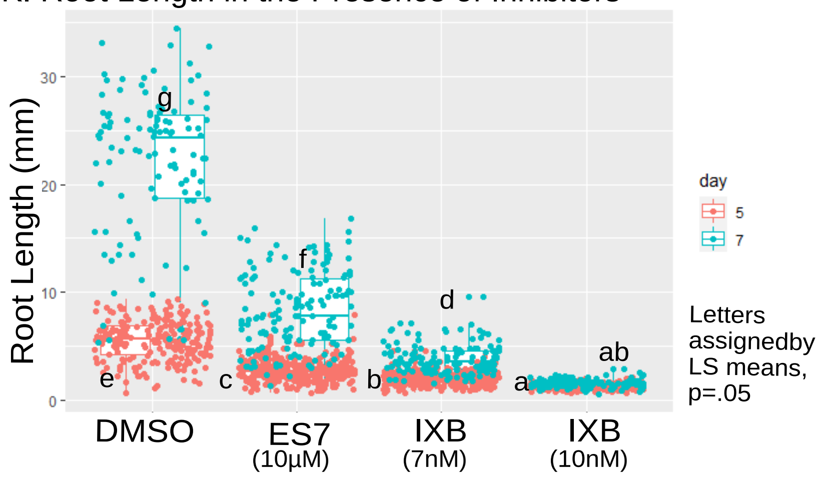

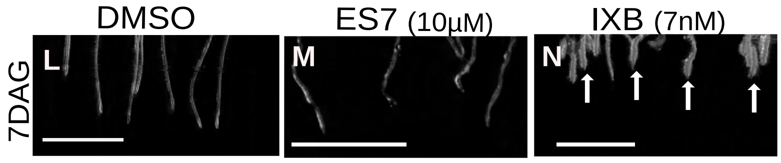

Supplemental Figure S12. Effect of isoxaben (IXB) and Endosidin7 (ES7) on cellular organization and root growth.
A – I, Cellular organization of Arabidopsis root tips in 7 nM IXB and 10 µM ES7.
Arabidopsis root tips from 5 day old seedlings grown under chemical treatments.
A – C, DMSO treated seedlings display regular cellular organization without cytokinetic defects.
D – F, Treatment with ES7 leads to cytokinetic defects in the form of discontinuous cell walls, indicated by a star.
G – I, Treatment with IXB leads to a swollen cell phenotype. The cytokinesis marker RABA2a is shown in green, while FM4-64 staining of plasma membrane is shown in magenta. Samples are single scans of live cell confocal imaging. Bars = 10 µm.
J, Quantification of the discontinuous cell wall phenotype showed a 27 % in ES7 treatment with no discernable phenotype in DMSO or IXB treatment. Data represent quantification of 5-10 seedlings per treatment
K, Germination of Arabidopsis seedlings in 7 nM IXB and 10 µM ES7 5 and 7 days after germination (DAG). The root growth inhibition is significantly higher under IXB treatment compared to ES7. Letters assigned by LS means, p = 0.05. (5 day/ 7 days). DMSO 7 DAG n = 212, DMSO 5 DAG n =110, ES7 5 DAG n = 356, ES7 7 DAG n = 145, IXB 7 nM 5 DAG n = 324, IXB 7 nM 5DAG n =136, IXB 5 DAG 10 nM n = 289, IXB 7 DAG n=125. Individual data points of root length were plotted in box-whisker plot. Boxes indicate the median and interquartile range. Whiskers show 1.5 times the interquartile range.
L- N, Root tips of Arabidopsis seedlings 7 days after germination in media supplemented with DMSO (**L**), 10 µM ES7 (**M**) and 7 nM IXB (**N**). Note the prominent root swelling in IXB compared to the other treatments.

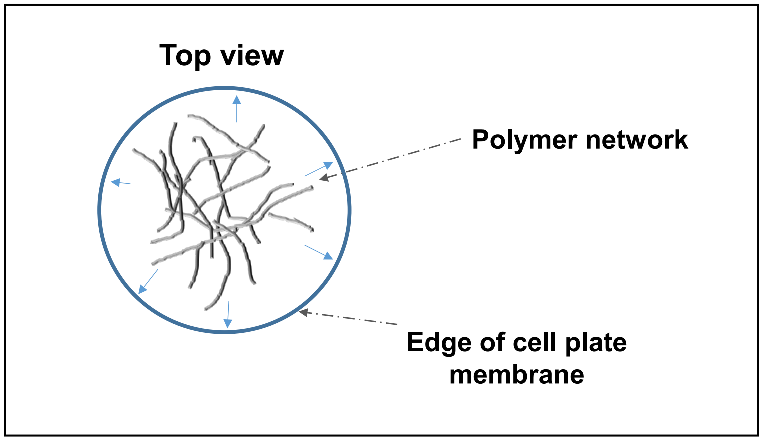

Supplemental Figure S13. **Proposed model of polymer deposition generating a two-dimensional spreading force.**
Growth of polymer as a 2D self-avoiding polymer exerts a radial outward areal pressure on the edge of the cell plate. The force acts within the plate only and thus includes cross-sectional area (not fenestrations).

Supplementary Information References

Abbena E, Salamon S, Gray A (2017) Modern Differential Geometry of Curves and Surfaces with Mathematica. CRC Press

Choksi R, Morandotti M, Veneroni M (2013) Global minimizers for axisymmetric multiphase membranes. Esaim-Control Optimisation and Calculus of Variations **19:** 1014-1029

Dimova R (2014) Recent developments in the field of bending rigidity measurements on membranes. Advances in Colloid and Interface Science **208:** 225-234

Fischer-Friedrich E, Hyman AA, Juelicher F, Mueller DJ, Helenius J (2014) Quantification of surface tension and internal pressure generated by single mitotic cells. Scientific Reports **4**

Helfrich W (1973) Elastic properties of lipid bilayers - theory and possible experiments. Zeitschrift Fur Naturforschung C-a Journal of Biosciences **C 28:** 693-703

Him JLK, Pelosi L, Chanzy H, Putaux JL, Bulone V (2001) Biosynthesis of (1 -> 3)-beta-D-glucan (callose) by detergent extracts of a microsomal fraction from Arabidopsis thaliana. European Journal of Biochemistry **268:** 4628-4638

Hu M, Briguglio JJ, Deserno M (2012) Determining the Gaussian Curvature Modulus of Lipid Membranes in Simulations. Biophysical Journal **102:** 1403-1410

Lazaro GR, Pagonabarraga I, Hernandez-Machado A (2015) Phase-field theories for mathematical modeling of biological membranes. Chemistry and Physics of Lipids **185:** 46-60

Lee JM (1997) Riemannian Manifolds. Springer-Verlag New York, Inc.

Mogilner A, (2003) Force Generation by Actin Polymerization II: The Elastic Ratchet and Tethered Filaments. Biophysical Journal **84:** 1591-1605

Park E, Diaz-Moreno SM, Davis DJ, Wilkop TE, Bulone V, Drakakaki G (2014) Endosidin 7 Specifically Arrests Late Cytokinesis and Inhibits Callose Biosynthesis, Revealing Distinct Trafficking Events during Cell Plate Maturation. Plant Physiology **165:** 1019-1034

Pelosi L, Imai T, Chanzy H, Heux L, Buhler E, Bulone V (2003) Structural and morphological diversity of (1-->3)-beta-D-glucans synthesized in vitro by enzymes from Saprolegnia monoïca. Comparison with a corresponding in vitro product from blackberry (Rubus fruticosus). Biochemistry **42:** 6264-6274

Rawicz W, Olbrich KC, McIntosh T, Needham D, Evans E (2000) Effect of chain length and unsaturation on elasticity of lipid bilayers. Biophysical Journal **79:** 328-339

Sarasij RC, Mayor S, Rao M (2007) Chirality-induced budding: A raft-mediated mechanism for endocytosis and morphology of caveolae? Biophysical Journal **92:** 3140-3158

Schneider CA, Rasband WS, Eliceiri KW (2012) NIH Image to ImageJ: 25 years of image analysis. Nat Methods **9:** 671-675

Schulmann N, Meyer H, Kreer T, Cavallo A, Johner A, Baschnagel J, Wittmer JP (2013) Strictly Two-Dimensional Self-Avoiding Walks: Density Crossover Scaling. Polymer Science Series C **55:** 181-211

van Oostende-Triplet C, Guillet D, Triplet T, Pandzic E, Wiseman PW, Geitmann A (2017) Vesicle Dynamics during Plant Cell Cytokinesis Reveals Distinct Developmental Phases. Plant Physiol **174:** 1544-1558

Worden N, Esteve VE, Domozych DS, Drakakaki G (2015) Using chemical genomics to study cell wall formation and cell growth in Arabidopsis thaliana and Penium margaritaceum. Methods Mol Biol **1242:** 23-39
