# Supplementary figures and images for: A biophysical model for plant cell plate maturation based on the contribution of a spreading force
Source: Plant Physiol. 2021 Nov 27;188(2):795–806. doi: 10.1093/plphys/kiab552 (PMC8825336; doi:10.1093/plphys/kiab552)

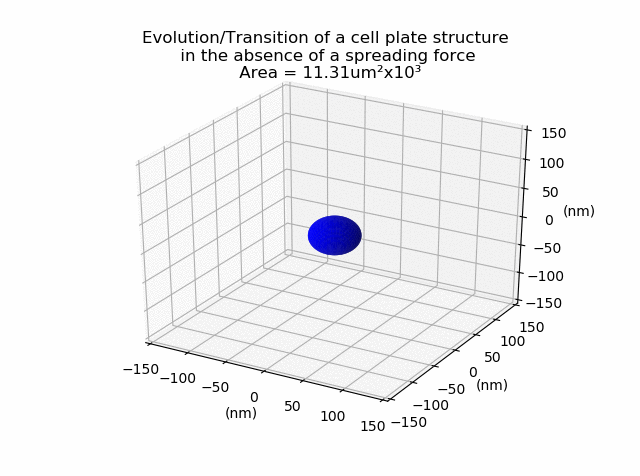

Supplement: kiab552_Supplementary_Data [file kiab552_supplementary_data.zip › SVideo1.gif]

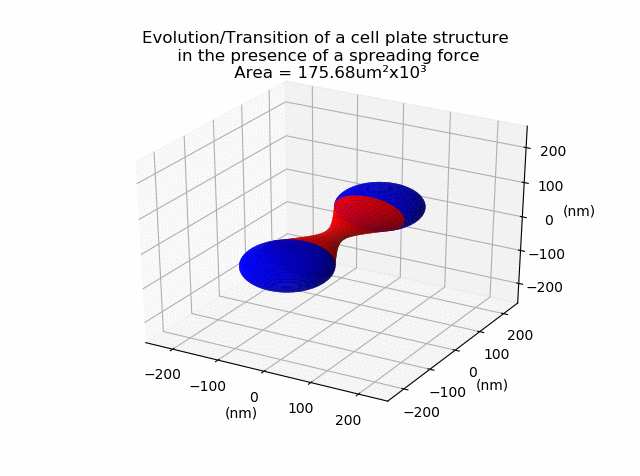

Supplement: kiab552_Supplementary_Data [file kiab552_supplementary_data.zip › SVideo2.gif]
